# Long-term efficacy and safety of inotersen for hereditary transthyretin amyloidosis: NEURO-TTR open-label extension 3-year update

**DOI:** 10.1007/s00415-022-11276-8

**Published:** 2022-07-31

**Authors:** Thomas H. Brannagan, Teresa Coelho, Annabel K. Wang, Michael J. Polydefkis, Peter J. Dyck, John L. Berk, Brian Drachman, Peter Gorevic, Carol Whelan, Isabel Conceição, Violaine Plante-Bordeneuve, Giampaolo Merlini, Laura Obici, Josep Maria Campistol Plana, Josep Gamez, Arnt V. Kristen, Anna Mazzeo, Luca Gentile, Arvind Narayana, Kemi Olugemo, Peter Aquino, Merrill D. Benson, Morie Gertz, for the NEURO-T. T. R. Open-Label Extension Investigators

**Affiliations:** 1 Department of Neurology, Columbia University Medical Center, 710 West 168th Street, New York, NY 10032 USA; 2 Centro Hospitalar Universitário do Porto, Porto, Portugal; 3 Neuromuscular Diagnostic Laboratory, University of California, Irvine, Orange, CA USA; 4 Department of Neurology, Johns Hopkins University, Baltimore, MD USA; 5 Neurology and General Internal Medicine, Mayo Clinic, Rochester, MN USA; 6 Amyloid Clinic, Boston University, Boston, MA USA; 7 Cardiovascular Medicine, Penn Presbyterian Medical Center, Philadelphia, PA USA; 8 Department of Rheumatology, Mount Sinai Medical Center, New York, NY USA; 9 Cardiology, National Amyloidosis Centre, Royal Free Hospital, London, UK; 10 Department of Neurology, CHULN, Hospital Santa Maria and Faculdade de Medicina, Universidade de Lisboa, Lisbon, Portugal; 11 CHU Henri Mondor, Creteil, France; 12 Amyloidosis Research and Treatment Center, IRCCS Fondazione Policlinico San Matteo, University of Pavia, Pavia, Italy; 13 Hospital Clinic, University of Barcelona, Institut d’Investigacions Biomèdiques August Pi i Sunyer, Barcelona, Spain; 14 Neurology Department, GMA Clinic, Autonomous University of Barcelona and European Reference Network on Rare Neuromuscular Diseases (ERN EURO-NMD), Barcelona, Spain; 15 Amyloidosis Center, Heidelberg University Hospital, Heidelberg, Germany; 16 Department of Clinical and Experimental Medicine, University of Messina, Messina, Italy; 17 Akcea Therapeutics, Boston, MA USA; 18 Department of Pathology and Laboratory Medicine, Indiana University School of Medicine, Indianapolis, IN USA

**Keywords:** Clinical trial, Familial amyloid polyneuropathy, Inotersen, Hereditary transthyretin amyloidosis, Peripheral neuropathies, Polyneuropathy

## Abstract

**Background:**

Hereditary transthyretin amyloidosis (hATTR/ATTRv) results from the deposition of misfolded transthyretin (TTR) throughout the body, including peripheral nerves. Inotersen, an antisense oligonucleotide inhibitor of hepatic TTR production, demonstrated a favorable efficacy and safety profile in patients with the polyneuropathy associated with hATTR in the NEURO-TTR (NCT01737398) study. We report longer-term efficacy and safety data for inotersen, with a median treatment exposure of 3 years.

**Methods:**

Patients who satisfactorily completed NEURO-TTR were enrolled in its open-label extension (OLE) study. Efficacy assessments included the modified Neuropathy Impairment Score + 7 (mNIS + 7), Norfolk Quality of Life–Diabetic Neuropathy (Norfolk QoL-DN) questionnaire total score, and the Short Form 36 (SF-36v2) Health Survey Physical Component Summary score. Safety and tolerability were also assessed. Efficacy is reported for patients living in Europe and North America (this cohort completed the study approximately 9 months before the remaining group of patients outside these regions); safety is reported for the full safety dataset, comprising patients living in Europe, North America, and Latin America/Australasia. This study is registered with ClinicalTrials.gov, identifier NCT02175004.

**Results:**

In the Europe and North America cohort of the NEURO-TTR study, 113/141 patients (80.1%) completed the study, and 109 patients participated in the OLE study. A total of 70 patients continued to receive inotersen (inotersen–inotersen) and 39 switched from placebo to inotersen (placebo–inotersen). The placebo–inotersen group demonstrated sustained improvement in neurological disease progression as measured by mNIS + 7, compared with predicted worsening based on projection of the NEURO-TTR placebo data (estimated natural history). The inotersen–inotersen group demonstrated sustained benefit, as measured by mNIS + 7, Norfolk QoL-DN, and SF-36v2, compared with estimated natural history as well as compared with the placebo–inotersen group. With a maximum exposure of 6.2 years, inotersen was not associated with any additional safety concerns or increased toxicity in the OLE study. Platelet and renal monitoring were effective in reducing the risk of severe adverse events in the OLE study.

**Conclusion:**

Inotersen treatment for > 3 years slowed progression of the polyneuropathy associated with hATTR, and no new safety signals were observed.

**Supplementary Information:**

The online version contains supplementary material available at 10.1007/s00415-022-11276-8.

## Introduction

Hereditary transthyretin amyloidosis (hATTR or ATTRv [variant]) is a rapidly progressive, debilitating, and ultimately fatal disease resulting from the accumulation of transthyretin (TTR) amyloid fibrils in peripheral nerves and various other organs and tissues throughout the body [1]. It is caused by mutations in the *TTR* gene that are thought to induce changes in the protein’s normally tetrameric structure, such that it dissociates to monomer subunits which then misfolds and have a greater propensity for aggregation and the formation of amyloid fibrils [1, 2]. In patients with hATTR, systemic deposition of amyloid fibrils causes debilitating sensorimotor peripheral neuropathy and autonomic neuropathy, cardiomyopathy with associated heart failure, as well as ocular and renal disturbances [1, 3–6]. hATTR can manifest with a predominant polyneuropathy or cardiomyopathy phenotype, or a mixture of both, depending on the *TTR* variant [5, 7, 8].

Early signs/symptoms of hATTR include pain and loss of temperature sensation, along with digestive problems and genitourinary dysfunction [9, 10]. As the disease course progresses, patients experience muscle weakness and balance abnormalities [10]. If left untreated, polyneuropathy associated with hATTR has a rapidly progressive course, with associated walking difficulties and loss of ambulation as well as malnutrition [11].

Patients experience substantial disease burden and a rapid decline in quality of life (QoL), with progressive neuropathy, cardiomyopathy, and psychosocial manifestations affecting every aspect of their lives [11–15]. Therefore, early diagnosis and implementation of treatment is imperative to slow progression of the disease [16]. In the global, randomized, double-blind, placebo-controlled pivotal NEURO-TTR study in adults with the polyneuropathy of hATTR, inotersen slowed progression of polyneuropathy and deterioration in patients’ QoL [17]. The occurrence of glomerulonephritis and thrombocytopenia (platelet count decreases of < 25 × 10^9^/L) in three patients each following inotersen treatment prompted initiation of enhanced safety monitoring, which has been effective in mitigating future risk of these events. We present the efficacy and safety data from the open-label extension (OLE) study of NEURO-TTR, with an overall median treatment exposure of ~ 3 years and maximum exposure of 5 years.

## Methods

### Study design

This OLE study (NCT02175004) of the global, randomized, double-blind, placebo-controlled pivotal NEURO-TTR trial (NCT01737398) [17] consisted of a ≤ 4-week screening period, a treatment period of up to 260 weeks, and a 12-week post-treatment evaluation period. The study design and inclusion/exclusion criteria for the NEURO-TTR [17] and NEURO-TTR OLE studies have been previously published [18]. Briefly, patients with stage 1 or 2 polyneuropathy associated with hATTR and a Neuropathy Impairment Score ≥ 10 and ≤ 130 who satisfactorily completed the randomized NEURO-TTR study could enter the OLE study and receive 300 mg inotersen once weekly via subcutaneous injection for up to 260 weeks (5 years). One patient who did not complete the NEURO-TTR study was nonetheless allowed to enter the OLE. Patients receiving inotersen in NEURO-TTR continued to receive inotersen in the OLE study and are hereafter referred to as the inotersen–inotersen group; patients receiving placebo in NEURO-TTR switched to inotersen in the OLE study and are hereafter referred to as the placebo–inotersen group.

The trial protocol for the OLE study was approved by the relevant institutional review boards or local ethics committees and regulatory authorities. The trial was conducted in accordance with Good Clinical Practice guidelines of the International Conference on Harmonisation and the principles of the Declaration of Helsinki. All patients provided written informed consent to participate in the study.

### Outcomes

The primary objective of the OLE study was the evaluation of the safety of extended dosing with inotersen. Safety assessments included the incidence of adverse events (AEs) and treatment-emergent AEs (TEAEs) reported during the study per National Cancer Institute Common Terminology Criteria for Adverse Events, version 4.03. The change in TTR levels from baseline up to week 156 was determined during the study. Efficacy endpoints included change from baseline up to week 156 in the (a) modified Neuropathy Impairment Score + 7 composite score (mNIS + 7; range − 22.3 to 346.3, with higher scores indicating poorer function), (b) Norfolk Quality of Life–Diabetic Neuropathy questionnaire scores (Norfolk QoL-DN; range − 4 to 136, with higher scores indicating poorer QoL), and (c) 36-Item Short-Form Health Survey (SF-36) questionnaire scores (range, 0 to 100; lower score indicating worse QoL).

### Statistical analysis

The OLE baseline values were carried forward from the week 65 visit of the NEURO-TTR study for the SF-36 and from the week 66 visit for mNIS + 7 and QoL-DN measures. Responders were defined as patients whose change in score was less than the responder definition (RD) threshold for the mNIS + 7 and Norfolk QoL-DN measures and greater than the RD threshold for the SF-36; that is, their change in score did not indicate clinical worsening (i.e., responders included both patients with no clinically relevant change in scores plus patients who improved with treatment). Using data from the NEURO-TTR study, the RD thresholds the Norfolk QOL-DN (8.8 points) were estimated using both anchor-based and distribution-based methods and for the mNIS + 7 (12.2 points) were estimated using distribution-based methods only, because there were no appropriate anchor measures available [19]. The threshold for the SF-36 PCS (− 5 points) was based on studies from the literature that utilized both anchor-based and distribution-based methods [20–22]. For each estimated RD threshold, responder analyses compared the proportion of patients on the mNIS + 7, Norfolk QOL-DN, and SF-36 PCS between inotersen–inotersen and placebo–inotersen groups at each visit up to week 156.

As of July 28, 2020, efficacy was reported for the Europe and North America cohort that received ≥ 1 dose of inotersen in the OLE and had ≥ 1 post-baseline efficacy assessment (full analysis set). This cohort completed database lock approximately 9 months before the remaining group of patients in the Latin America/Australasia cohort. For safety analyses, data are reported for the full safety dataset, which comprises patients from Europe, North America, and Latin America/Australasia.

## Results

### Study population

A total of 139 (80%) patients completed the NEURO-TTR study, including 113 patients in the Europe and North America cohort [17]. Of the 139 patients who completed the NEURO-TTR study, 135 (97%) participated in the OLE study, of whom 109 patients comprised the Europe and North America cohort. A total of 70 patients in the Europe and North America cohort continued to receive inotersen (inotersen–inotersen) and 39 switched from placebo to inotersen (placebo–inotersen) (Online Resource 1, Fig. 1). The primary reasons for treatment discontinuation are specified in Online Resource 1, Fig. 1. Of the 108 patients who discontinued the OLE study, 43 (39.8%) switched to a commercial drug (Tegsedi^®^): 25 patients from the inotersen–inotersen treatment group and 18 patients from the placebo–inotersen group. In the OLE study, baseline demographics and clinical characteristics were generally balanced between the inotersen–inotersen and placebo–inotersen groups (Table 1). A higher proportion of patients in the inotersen–inotersen group had a polyneuropathy disability score of I/II at baseline compared with the placebo–inotersen group (65.7% vs. 53.8%).

**Fig. 1:**
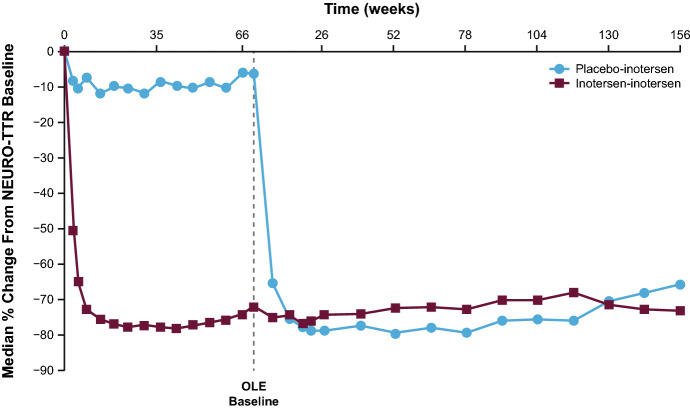
OLE median serum transthyretin (TTR) levels relative to NEURO-TTR baseline. The OLE baseline on the graph is noted as occurring 4 weeks after the end of the NEURO-TTR study because the maximum screening period in the OLE study was  generally 4 weeks. *OLE* open-label extension, *SE* standard error, *TTR* transthyretin

**Table 1:** OLE study baseline demographics and clinical characteristics (Europe and North America cohort)

Characteristic	Inotersen–inotersen (*n* = 70)	Placebo–inotersen (*n* = 39)
Age at OLE study screening, median (range), years	65 (58–70)	66 (59–75)
Male, *n* (%)	51 (72.9)	28 (71.8)
PND score at OLE study baseline,^a^ *n* (%)		
I/II	46 (65.7)	21 (53.8)
III/IV	22 (31.4)	17 (43.6)
V	2 (2.9)	1 (2.6)
Val30Met *TTR* mutation,^b^ *n* (%)	25 (35.7)	19 (48.7)
Prior TTR stabilizer use,^b,c^ *n* (%)	48 (68.6)	23 (59.0)
mNIS + 7 composite score at NEURO-TTR baseline,^d^ mean (SD)	79.9 (36.4)	77.4 (36.1)
mNIS + 7 composite score at OLE baseline,^e^ mean (SD)	85.3 (40.0)	102.7 (47.9)
Norfolk QoL-DN total score at NEURO-TTR baseline,^f^ mean (SD)	47.4 (27.4)	49.6 (28.1)
Norfolk QoL-DN total score at OLE baseline,^g^ mean (SD)	50.9 (29.1)	61.2 (31.5)
SF-36 score at NEURO-TTR baseline,^h^ mean (SD)	35.6 (8.6)	36.8 (9.5)
SF-36 score at OLE baseline,^i^ mean (SD)	34.6 (9.9)	33.4 (10.5)
Duration from onset of hATTR amyloidosis PN symptoms to OLE baseline, mean (SD), mo	81.6 (52.4)	87.1 (60.5)

*hATTR* hereditary transthyretin amyloidosis, *mNIS* + *7* modified Neuropathy Impairment Score + 7 neurophysiological tests composite score, *Norfolk QoL-DN* Norfolk Quality of Life–Diabetic Neuropathy questionnaire total score, *OLE* open-label extension, PN polyneuropathy, *PND* polyneuropathy disability, *SD* standard deviation, *TTR* transthyretin

^a^PND score is defined as I = sensory disturbances in limbs without motor impairment; II = difficulty walking without the need of a walking aid; III = 1 stick or 1 crutch required for walking; IV = 2 sticks or 2 crutches needed; V = wheelchair required or patient confined to bed

^b^Based on data entered in the electronic case report form at NEURO-TTR study entry

^c^Prior stabilizer use includes tafamidis and/or diflunisal

^d^NEURO-TTR baseline mNIS + 7 based on 67 inotersen–inotersen patients and 39 placebo–inotersen patients

^e^OLE baseline mNIS + 7 based on 66 inotersen–inotersen patients and 39 placebo–inotersen patients from the week 66 visit of the NEURO-TTR study

^f^NEURO-TTR baseline Norfolk QoL-DN based on 66 inotersen–inotersen patients and 39 placebo–inotersen patients

^g^OLE baseline Norfolk QoL-DN based on 65 inotersen–inotersen patients and 39 placebo–inotersen patients from the week 66 visit of the NEURO-TTR study

^h^NEURO-TTR baseline SF-36 scores based on 69 inotersen–inotersen patients and 39 placebo–inotersen patients

^i^OLE baseline SF-36 scores based on 67 inotersen–inotersen patients and 38 placebo–inotersen patients from week 65 visit of the NEURO-TTR study

With extended follow-up, median treatment exposure for the safety population in the OLE study was 987 days (range 1–1814 days), and the longest combined exposure of inotersen during the NEURO-TTR plus OLE study, measured as the time from the first dose to the last dose, was 2270 days (6.2 years). Median treatment exposure for the inotersen–inotersen and placebo–inotersen groups was 1063 days (1–1814 days) and 938 days (106–1728 days), respectively. Dose interruption and reasons for dose interruption are provided in Online Resource 2.

### Pharmacodynamics

In the OLE study, reduction in TTR level was sustained in the inotersen–inotersen group to week 156, reaching a median nadir of 73% relative to NEURO-TTR baseline (Fig. 1). In the placebo–inotersen group, there was a notable reduction in TTR level by OLE week 7, when the first measurement was taken (Fig. 1). The reduction continued through week 156 in the OLE study, reaching a median nadir of 66% relative to OLE baseline.

### Efficacy

#### Neuropathy impairment (mNIS + 7)

**Fig. 2:**
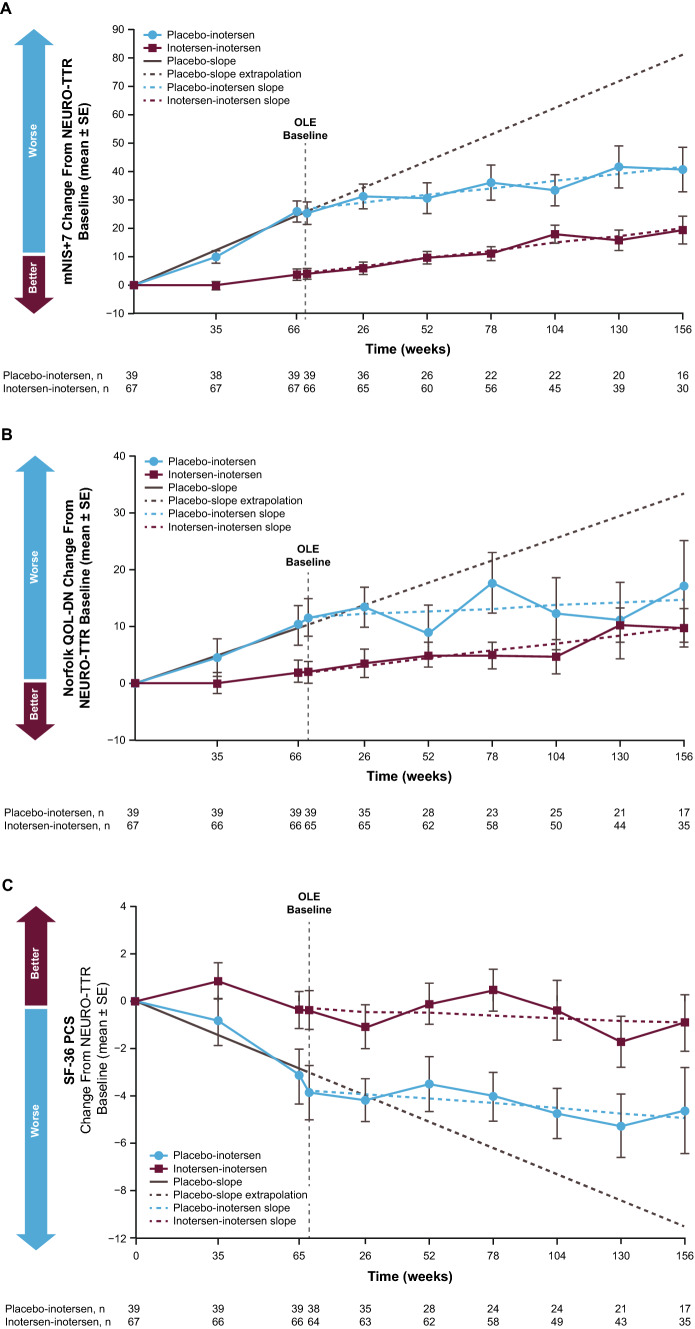
Mean change from NEURO-TTR baseline to OLE week 156 in efficacy measures. Mean (± SE) change from NEURO-TTR baseline in **A** the Modified Neuropathy Impairment Score + 7 (mNIS + 7); **B** the Norfolk Quality of Life–Diabetic Neuropathy Questionnaire Total Score (QoL-DN); **C** the 36-Item Short-Form Health Survey, version 2 (SF-36), Physical Component Summary score (PCS). The vertical dashed line represents OLE baseline (OLE week 0). Sample sizes for each time point and treatment group are indicated under the figure. The OLE baseline values were carried forward from the week 65 visit of the NEURO-TTR study for the SF-36 and from the week 66 visit for mNIS + 7 and QoL-DN measures. The OLE baseline on the graph is noted as occurring 4 weeks after the end of the NEURO-TTR study because the maximum screening period in the OLE study was generally 4 weeks. *OLE*, open-label extension, *SE*, standard error

The inotersen–inotersen group demonstrated sustained benefit, as measured by mNIS + 7, where higher scores are indicative of poorer neurological function, compared with the placebo–inotersen group (Fig. 2A). The mean change from NEURO-TTR study baseline to OLE study baseline, as well as weeks 52, 104, and 156 for the mNIS + 7, was 4.0, 9.5, 17.8, and 19.2, respectively, in the inotersen–inotersen group. The placebo–inotersen group demonstrated sustained improvement in neurological disease progression, compared with predicted worsening based on a projection of the placebo data (estimated natural history). The corresponding mean change from NEURO-TTR baseline for the placebo–inotersen group was 25.3, 30.5, 33.4, and 40.6. Compared with projected natural history data, the mean change from NEURO-TTR baseline for mNIS + 7 was 62.2 points lower in the inotersen–inotersen group and 40.7 points lower in the placebo–inotersen group at week 156.

**Fig. 3:**
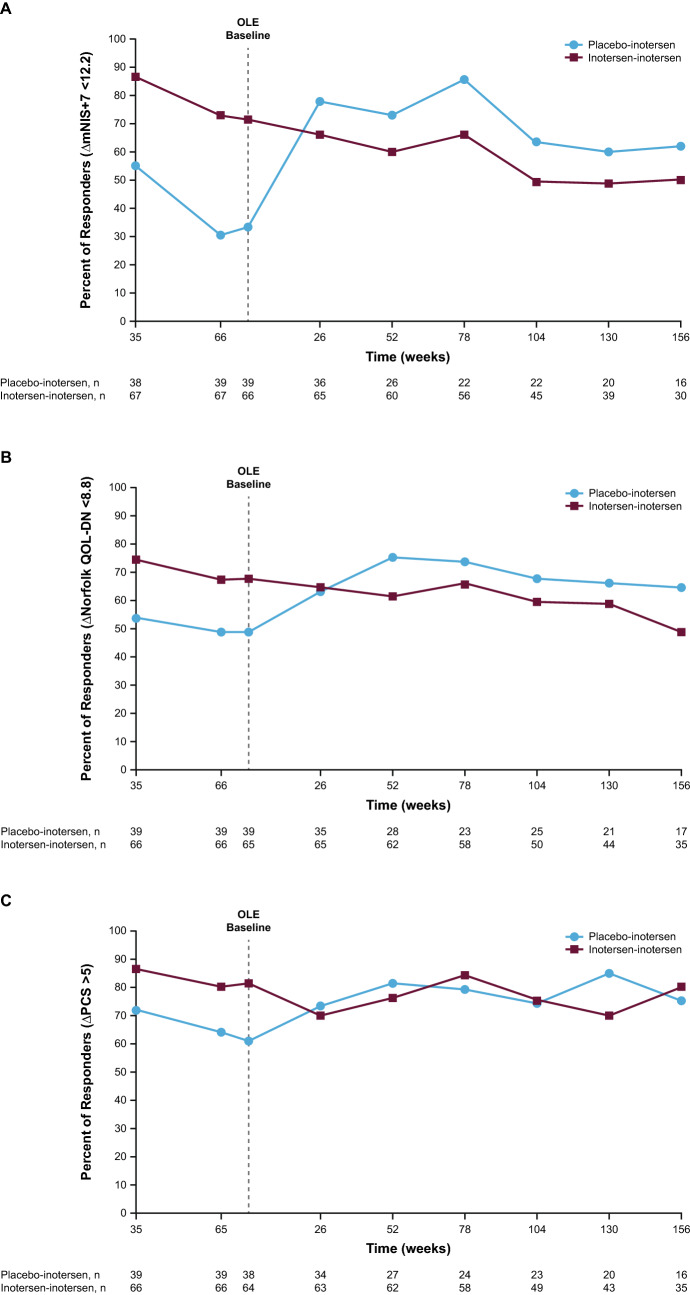
Responder analyses for the NEURO-TTR Europe and North America cohort. Data are presented for **A** the Modified Neuropathy Impairment Score + 7 Neurophysiological Tests Composite Score (mNIS + 7), **B** the Norfolk Quality of Life–Diabetic Neuropathy Questionnaire Total Score (QoL-DN), and **C** the 36-Item Short-Form Health Survey, version 2 (SF-36), Physical Component Summary score (PCS). A patient was classified as a responder at various time points through week 156 if they showed no clinically meaningful progression based on the applicable threshold and had a recorded measurement at baseline and at the relevant time point. The OLE baseline on the graph is noted as occurring 4 weeks after the end of the NEURO-TTR study because the maximum screening period in the OLE study was generally 4 weeks. **A** For the inotersen–inotersen group, responders at all visits are based on change < 12.2 points from NEURO-TTR baseline. For the placebo–inotersen group, responders at NEURO-TTR week 35 and week 66 visits and OLE baseline visits are based on change < 12.2 points from NEURO-TTR baseline, while responders at all post-baseline OLE visits are based on change < 12.2 points from OLE baseline. **B** For the inotersen–inotersen group, responders at all visits are based on change < 8.8 points from NEURO-TTR baseline. For the placebo–inotersen group, responders at NEURO-TTR week 35 and week 66 visits and OLE baseline visits are based on change < 8.8 points from NEURO-TTR baseline, while responders at all post-baseline OLE visits are based on change < 8.8 points from OLE baseline. **C** For the inotersen–inotersen group, responders at all visits are based on change > –5 points from NEURO-TTR baseline. For the placebo–inotersen group, responders at NEURO-TTR week 35 and week 66 visits and OLE baseline visits are based on change > –5 points from NEURO-TTR baseline, while responders at all post-baseline OLE visits are based on change > –5 points from OLE baseline. *OLE*, open-label extension, *RD*, responder definition

Using a responder definition of a change in the mNIS + 7 score of < 12.2 points from their NEURO-TTR baseline, 60%, 49%, and 50% of patients in the inotersen–inotersen group improved at 52, 104, and 156 weeks of treatment in the OLE (Fig. 3A). Similarly, 73%, 64%, and 63% of placebo–inotersen patients had a change in mNIS + 7 score from their OLE baseline of < 12.2 points at 52, 104, and 156 weeks of treatment in the OLE (Fig. 3A). Improvement of mNIS + 7 score also was observed in the inotersen–inotersen and placebo–inotersen groups as indicated by a < 0.0-point change (Online Resource 3).

The improvement in mNIS + 7 compared with natural history was mostly maintained irrespective of disease stage (except for in the placebo–inotersen stage 2 group), genetic mutations (p.Val50Met [V30M] vs. no p.Val50Met), and prior treatment (with or without stabilizers) (Online Resource 4). Across all three baseline characteristics, there was generally a clear separation between the inotersen–inotersen group and the placebo–inotersen group.

#### Neuropathy-related QoL (Norfolk QoL-DN)

The inotersen–inotersen group demonstrated sustained benefit compared with the placebo–inotersen group, as measured by the Norfolk QoL-DN questionnaire in which higher scores are also indicative of poorer function (Fig. 2B). For the inotersen–inotersen group, the mean changes in the Norfolk QoL-DN from NEURO-TTR baseline to OLE baseline, 52, 104, and 156 weeks were 1.9, 5.1, 4.7, and 9.8, respectively. The placebo–inotersen group demonstrated stabilization in neuropathy-related QoL. The mean changes in Norfolk QoL-DN from NEURO-TTR baseline to OLE baseline and 52, 104, and 156 weeks were 11.6, 9.0, 12.3, and 17.3, respectively. Relative to the placebo-slope extrapolation, the mean change from NEURO-TTR baseline to OLE week 156 was 23.5 points lower in the inotersen–inotersen group and 15.9 points lower in the placebo–inotersen group.

Using a responder definition of a change of < 8.8 points in the Norfolk QoL-DN relative to NEURO-TTR baseline, improvement occurred in 61%, 60%, and 49% of patients in the inotersen–inotersen group after 52, 104, and 156 weeks of cumulative treatment during the OLE study (Fig. 3B). At the same time points, improvement in the Norfolk QoL-DN occurred in 75%, 68%, and 65% of patients, respectively (Fig. 3B) in the placebo–inotersen group, relative to OLE study baseline. Improvement of Norfolk QoL-DN was also observed for both inotersen–inotersen and placebo–inotersen groups as indicated by a < 0.0-point change (Online Resource 3).

The improvement in Norfolk QoL-DN compared with natural history was maintained for the inotersen–inotersen and placebo–inotersen groups for stage 1 disease (but not stage 2 disease), those both with and without a V30M mutation, and those receiving prior treatment (with or without stabilizers) (Online Resource 4). Across these three baseline characteristics, there was no clear separation over time between the inotersen–inotersen and placebo–inotersen groups.

#### Health-related QoL (SF-36 PCS)

The SF-36 is a non-disease-specific health questionnaire that assesses the patient’s perceived functional health, and higher scores are indicative of better function. As seen in the inotersen–inotersen group, early initiation of inotersen led to sustained benefit in health-related QoL compared with the placebo–inotersen group (Fig. 2C). For the inotersen–inotersen group, the mean change from NEURO-TTR baseline to OLE baseline and 52, 104, and 156 weeks for the SF-36 PCS was − 0.4, − 0.1, − 0.4, and − 0.9, respectively. For the placebo–inotersen group, the mean change was − 3.9, − 3.5, − 4.8, and − 4.6, respectively. Relative to the placebo-slope extrapolation, at week 156 of the OLE, SF-36 PCS scores were 8.6 points higher in the inotersen–inotersen group and 4.9 points higher in the placebo–inotersen group.

Using a responder definition of a change of − 5 points relative to the NEURO-TTR baseline, the percentage of responders based on the SF-36 PCS was 76%, 76%, and 80% of patients in the inotersen–inotersen group after 52, 104, and 156 weeks of cumulative treatment during the OLE study (Fig. 3C). At similar time points for patients in the placebo–inotersen group, the percentage of responders for SF-36 PCS was 81%, 74%, and 75% (Fig. 3C) relative to OLE study baseline. Improvement in SF-36 PCS was also observed for both the inotersen–inotersen and placebo–inotersen groups as indicated by a > 0.0-point change (Online Resource 3).

The improvement in SF-36 PCS compared with natural history was maintained for the inotersen–inotersen and placebo–inotersen groups irrespective of genetics (V30M mutation vs. no V30M mutation), disease stage (stage 1 vs. stage 2), and prior treatment (with or without stabilizers) (Online Resource 4). Across these three baseline characteristics, there was no clear separation over time between the inotersen–inotersen and placebo–inotersen groups.

### Safety and tolerability

Safety of inotersen in the OLE study is summarized in Table 2. The most common (≥ 10%) TEAEs across all patients in the OLE were thrombocytopenia (28.1%), diarrhea (25.9%), nausea (25.2%), urinary tract infection (24.2%), fall (22.2%), fatigue (20.0%), vomiting (20.0%), chills (17.8%), peripheral edema (17.0%), injection site pain (13.3%), injection site erythema (11.9%), constipation (11.9%), syncope (11.1%), dyspnea (11.1%), headache (11.1%), pyrexia (10.4%), and anemia (10.4%).

**Table 2:** Summary of treatment-emergent adverse events in the NEURO-TTR OLE study^a^

Event, *n* (%)	Total (*n* = 135)
Treatment exposure^b^ during OLE study, median (range), days	987 (1–1814)
Any TEAEs	132 (97.8)
TEAEs related to study treatment	99 (73.3)
TEAEs leading to permanent discontinuation of inotersen	30 (22.2)
TEAEs leading to dose reduction	13 (9.6)
TEAEs leading to dose interruption/delay	66 (48.9)
Serious TEAEs	64 (47.4)
Serious TEAEs related to study treatment	6 (4.4)
Fatal TEAEs	16 (11.9)
Fatal TEAEs related to study treatment	0

*OLE* open-label extension, *TEAE* treatment-emergent adverse event

^a^Represents all patients from NEURO-TTR OLE

^b^Includes time on drug only; excludes dose holidays

TEAEs leading to dose interruption, dose reduction, or study drug delay occurred in 79 (58.5%) patients. Overall, 30 (22.2%) patients discontinued because of a TEAE: 23 (27.1%) patients in the inotersen–inotersen group (thrombocytopenia [*n* = 4], glomerulonephritis [*n* = 1]) and seven (14.0%) patients in the placebo–inotersen group.

A total of 64 (47.4%) patients experienced a serious TEAE: 46 (54.1%) patients in the inotersen–inotersen group and 18 (36.0%) patients in the placebo–inotersen group. Few were considered related to inotersen (*n* = 6; 4.4%); these included thrombocytopenia (*n* = 2 in the inotersen–inotersen group), nausea (*n* = 1 in the placebo–inotersen group), chills (*n* = 1 in the inotersen–inotersen group), liver disorder (*n* = 1 in the inotersen–inotersen group), and hypertension (*n* = 1 in the inotersen–inotersen group). Study drug was interrupted for both events of thrombocytopenia (platelet count nadir of 32 × 10^9^/L [day 350] and 43 × 10^9^/L [day 519], respectively); neither patient permanently discontinued study drug nor withdrew from the study due to these events of thrombocytopenia. The declines in platelet count were reversed on interruption of dosing and the events were not associated with bleeding. For the event of nausea, the drug dose was interrupted, and the event recovered/resolved. For the event of hypertension, the drug was withdrawn and the event recovered/resolved. The event of chills was recovered/resolved without any changes to drug dose. For the event of liver disorder, elevations were predominantly seen in aspartate aminotransferase (AST) (less than 3 × upper limit of normal [ULN]) and alkaline phosphatase (less than 2 × ULN), with no increase in total bilirubin levels. Partial resolution of the enzyme elevations was observed during continued inotersen treatment and complete resolution occurred after discontinuation of treatment. Liver biopsy showed evidence of congestion thought to be secondary to transient hepatic venous outflow obstruction rather than hepatotoxicity.

Overall, 16 (11.9%) patients died (Table 2) in the OLE study, and none of these deaths were considered related to treatment.

#### Thrombocytopenia and glomerulonephritis

Platelet and renal monitoring were effective in the OLE study. In the safety population, 38% (32/85) of the inotersen–inotersen group and 52% (26/50) of the placebo–inotersen group had a dose interruption due to platelet monitoring rules. In the OLE study, 50% (25/50) of patients in the placebo–inotersen group and 48% (41/85) of patients in the inotersen–inotersen group experienced confirmed platelet count decreases of < 100 × 10^9^/L. Nonetheless, grade 4 platelet count decrease was reported in none of the patients in either group (Online Resource 5, Fig. 5). In addition, no cases of acute glomerulonephritis were reported in the two groups.

## Discussion

Currently, patients with the polyneuropathy associated with hATTR have an estimated life expectancy of 5–10 years from symptom onset [23]. Peripheral nerve function in particular has been shown to deteriorate more rapidly in patients with the polyneuropathy associated with hATTR relative to other peripheral neuropathies [23]. Therefore, effective treatment options are crucial to slow down disease progression and improve patients’ QoL [12].

The results of this OLE study confirm that long-term exposure to inotersen resulted in continued efficacy after 3 years, with no additional safety concerns or signs of increased toxicity. Mean mNIS + 7 and Norfolk QoL-DN scores were relatively similar at NEURO-TTR and OLE study baseline for the inotersen–inotersen group; however, scores for the placebo–inotersen group indicated more severe disease at OLE study baseline because of disease progression while on placebo during the NEURO-TTR study.

The progression of polyneuropathy was substantially delayed in patients treated with inotersen versus predicted natural history over a 3-year follow-up. Importantly, patients who initiated treatment with inotersen earlier derived a greater benefit with respect to neurological impairment and QoL when compared with patients who received delayed treatment with inotersen (i.e., those who switched from placebo to inotersen approximately 15 months later). A previous analysis of specific aspects of QoL from the OLE showed that treatment with inotersen stabilized most aspects of QoL for up to 3 years including activities of daily living and physical and social functioning. Moreover, the gap in QoL between those who had previously received inotersen or placebo in NEURO-TTR did not close by week 104 of the OLE, indicating the importance of early treatment for maintaining HRQoL in patients with hATTR [24]. Together, these results confirm that early diagnosis and treatment are essential for maximal benefit. Delayed treatment provides benefit by slowing disease progression and improving QoL compared with natural history.

The emerging availability of effective therapeutic options for the polyneuropathy associated with hATTR has enhanced awareness and recognition of this debilitating disease and promoted expeditious implementation of treatment, even in patients with mild disease.

Enhanced monitoring has reduced the risk of severe thrombocytopenia and acute glomerulonephritis. With a maximum exposure of up to 6.2 years, inotersen was not associated with any additional safety concerns or increased toxicity in the OLE study. Platelet and renal monitoring have been effective in the OLE study: among all patients, there were no cases of grade 4 platelet count decrease or acute glomerulonephritis.

Limitations of the current study include its open-label design with no placebo control, which could influence patient responses to QoL assessments and, to a much lesser extent, mNIS + 7 assessments. Next, inferential statistics were not completed, and the results are thus qualitative. In addition, efficacy analyses included a subgroup of the overall OLE population (albeit a fairly large subgroup, consisting of 81% of study participants). Thus, subgroup efficacy analyses based on certain baseline characteristics are difficult to interpret. In addition, all OLE studies are associated with “survivor bias,” as only data on patients continuously enrolled can be analyzed; OLE analyses generally exclude patients who drop out and may have suffered different outcomes than the continuing cohort, depending on the reason for dropout. Nonetheless, the results of this study are important in determining the long-term efficacy and safety of inotersen, even under imperfect conditions.

In the NEURO-TTR OLE study, extended treatment with inotersen for over 3 years slowed progression of the polyneuropathy associated with hATTR and maintained QoL in patients, with greater benefit observed in patients who initiated inotersen earlier versus patients with delayed treatment initiation. These long-term results further highlight the importance of early initiation of treatment. Grade 4 thrombocytopenia and acute glomerulonephritis were not reported with enhanced monitoring during the OLE study.

## Supplementary Information

Below is the link to the electronic supplementary material.

Supplementary file1 (DOCX 2743 KB)

## Data Availability

Data that underlie the results reported in this article and the respective individual participant data will not be shared.

## References

[1] Manganelli F, Fabrizi GM, Luigetti M, Mandich P, Mazzeo A, Pareyson D (2020). Hereditary transthyretin amyloidosis overview. Neurol Sci.

[2] Plante-Bordeneuve V, Said G (2011). Familial amyloid polyneuropathy. Lancet Neurol.

[3] Gertz MA, Benson MD, Dyck PJ, Grogan M, Coelho T, Cruz M, Berk JL, Plante-Bordeneuve V, Schmidt HHJ, Merlini G (2015). Diagnosis, rognosis, and therapy of transthyretin amyloidosis. J Am Coll Cardiol.

[4] Gertz MA (2017). Hereditary ATTR amyloidosis: burden of illness and diagnostic challenges. Am J Manag Care.

[5] Coelho T, Maurer MS, Suhr OB (2013). THAOS—The Transthyretin Amyloidosis Outcomes Survey: initial report on clinical manifestations in patients with hereditary and wild-type transthyretin amyloidosis. Curr Med Res Opin.

[6] Maurer MS, Hanna M, Grogan M, Dispenzieri A, Witteles R, Drachman B, Judge DP, Lenihan DJ, Gottlieb SS, Shah SJ, Steidley DE, Ventura H, Murali S, Silver MA, Jacoby D, Fedson S, Hummel SL, Kristen AV, Damy T, Planté-Bordeneuve V, Coelho T, Mundayat R, Suhr OB, Waddington Cruz M, Rapezzi C, Investigators THAOS (2016). Genotype and phenotype of transthyretin cardiac amyloidosis: THAOS (Transthyretin Amyloid Outcome Survey). J Am Coll Cardiol.

[7] Sekijima Y (2001) Hereditary transthyretin amyloidosis synonyms: familial amyloid polyneuropathy, familial transthyretin amyloidosis, hereditary ATTR amyloidosis. In: Adam MP, Ardinger HH, Pagon RA, et al (eds) Gene reviews. Seattle, WA: University of Washington. https://www.ncbi.nlm.nih.gov/books/NBK1194/. (**Accessed 14 Mar 2022**)

[8] Gertz MA, Mauermann ML, Grogan M, Coelho T (2019). Advances in the treatment of hereditary transthyretin amyloidosis: a review. Brain Behav.

[9] Dyck PJ, Lambert EH (1969). Dissociated sensation in amyloidosis. Compound action potential, quantitative histologic and teased-fiber, and electron microscopic studies of sural nerve biopsies. Arch Neurol.

[10] Adams D, Ando Y, Beirão JM, Coelho T, Gertz MA, Gillmore JD, Hawkins PN, Lousada I, Suhr OB, Merlini G (2021). Expert consensus recommendations to improve diagnosis of ATTR amyloidosis with polyneuropathy. J Neurol.

[11] Inês M, Coelho T, Conceição I, Ferreira L, de Carvalho M, Costa J (2020). Health-related quality of life in hereditary transthyretin amyloidosis polyneuropathy: a prospective, observational study. Orphanet J Rare Dis.

[12] Lane T, Bangova A, Fontana M, Hutt DF, Strehina SG, Whelan CJ, Hawkins PN, Gillmore JD (2015). Quality of life in ATTR amyloidosis. Orphanet J Rare Dis.

[13] Stewart M, Shaffer S, Murphy B, Loftus J, Alvir J, Cicchetti M, Lenderking WR (2018). Characterizing the high disease burden of transthyretin amyloidosis for patients and caregivers. Neurol Ther.

[14] Wixner J, Mundayat R, Karayal ON, Anan I, Karling P, Suhr OB, THAOS investigators (2014). THAOS: gastrointestinal manifestations of transthyretin amyloidosis—common complications of a rare disease. Orphanet J Rare Dis.

[15] Coelho T, Vinik A, Vinik EJ, Tripp T, Packman J, Grogan DR (2017). Clinical measures in transthyretin familial amyloid polyneuropathy. Muscle Nerve.

[16] Adams D, Suhr OB, Hund E, Obici L, Tournev I, Campistol JM, Slama MS, Hazenberg BP, Coelho T, European Network for TTR-FAP (ATTReuNET) (2016). First European consensus for diagnosis, management, and treatment of transthyretin familial amyloid polyneuropathy. Curr Opin Neurol.

[17] Benson MD, Waddington-Cruz M, Berk JL, Polydefkis M, Dyck PJ, Wang AK, Planté-Bordeneuve V, Barroso FA, Merlini G, Obici L, Scheinberg M, Brannagan TH, Litchy WJ, Whelan C, Drachman BM, Adams D, Heitner SB, Conceição I, Schmidt HH, Vita G, Campistol JM, Gamez J, Gorevic PD, Gane E, Shah AM, Solomon SD, Monia BP, Hughes SG, Kwoh TJ, McEvoy BW, Jung SW, Baker BF, Ackermann EJ, Gertz MA, Coelho T (2018). Inotersen treatment for patients with hereditary transthyretin amyloidosis. N Engl J Med.

[18] Brannagan TH, Wang AK, Coelho T, Waddington Cruz M, Polydefkis MJ, Dyck PJ, Plante-Bordeneuve V, Berk JL, Barroso F, Merlini G, Conceição I, Hughes SG, Kwoh J, Jung SW, Guthrie S, Pollock M, Benson MD, Gertz M, NEURO-TTR open-label extension investigators (2020). Early data on long-term efficacy and safety of inotersen in patients with hereditary transthyretin amyloidosis: a 2-year update from the open-label extension of the NEURO-TTR trial. Eur J Neurol.

[19] Yarlas A, Lovley A, Brown D, Kosinski M, Vera-Llonch M (2022). Responder analysis for neuropathic impairment and quality-of-life assessment in patients with hereditary transthyretin amyloidosis with polyneuropathy in the NEURO-TTR study. J Neurol.

[20] Badhiwala JH, Witiw CD, Nassiri F, Akbar MA, Jaja B, Wilson JR, Fehlings MG (2018). Minimum clinically important difference in SF-36 scores for use in degenerative cervical myelopathy. Spine (Phila Pa 1976).

[21] Ogura K, Yakoub MA, Christ AB, Fujiwara T, Nikolic Z, Boland P, Healey JH (2020). What are the minimum clinically important differences in SF-36 scores in patients with orthopaedic oncologic conditions?. Clin Orthop Relat Res.

[22] Auffinger B, Lam S, Shen J, Thaci B, Roitberg BZ (2013). Usefulness of minimum clinically important difference for assessing patients with subaxial degenerative cervical spine disease: statistical versus substantial clinical benefit. Acta Neurochir (Wien).

[23] Lin X, Yarlas A, Vera-Llonch M, Baranwal N, Biber J, Brown D, Vogt B, Karam C (2021). Rate of neuropathic progression in hereditary transthyretin amyloidosis with polyneuropathy and other peripheral neuropathies: a systematic review and meta-analysis. BMC Neurol.

[24] Yarlas A, Lovley A, McCausland K, Brown D, Vera-Llonch M, Conceição I, Karam C, Khella S, Obici L, Waddington-Cruz M (2021). Early data on the long-term impact of inotersen on quality-of-life in patients with hereditary transthyretin amyloidosis polyneuropathy: open-label extension of NEURO-TTR. Neurol Ther.

