# Supplementary material for: Long-term efficacy and safety of inotersen for hereditary transthyretin amyloidosis: NEURO-TTR open-label extension 3-year update
Source: J Neurol. 2022 Jul 31;269(12):6416–27. doi: 10.1007/s00415-022-11276-8 (PMC9618524; doi:10.1007/s00415-022-11276-8)
Supplement: Supplementary file 1 — Supplementary file1 (DOCX 2743 KB) [file 415_2022_11276_MOESM1_ESM.docx]

**Long-term efficacy and safety of inotersen for hereditary transthyretin amyloidosis: NEURO-TTR open-label extension 3-year update**

*Journal of Neurology*

**Authors:** Thomas H. Brannagan III, Teresa Coelho, Annabel K. Wang, Michael J. Polydefkis, Peter J. Dyck, John L. Berk, Brian Drachman, Peter Gorevic, Carol Whelan, Isabel Conceição, Violaine Plante-Bordeneuve, Giampaolo Merlini, Laura Obici, Josep Maria Campistol Plana, Josep Gamez, Arnt Kristen, Anna Mazzeo, Luca Gentile, Arvind Narayana, Kemi Olugemo, Peter Aquino, Merrill D. Benson, Morie Gertz; for the NEURO-TTR Open-Label Extension Investigators*

*A complete list of NEURO-TTR Open-Label Extension investigators is provided in Online Resource 6.

**Corresponding Author:** Thomas H. Brannagan III, MD, Department of Neurology, Columbia University Medical Center, 710 West 168th Street, New York, NY 10032. Tel: (212) 305-0405; Fax: (212) 305-5396; email: [tb2325@cumc.columbia.edu](mailto:tb2325@cumc.columbia.edu)

# **Supplementary Material**

## **Online Resource 1.**

## Patient disposition


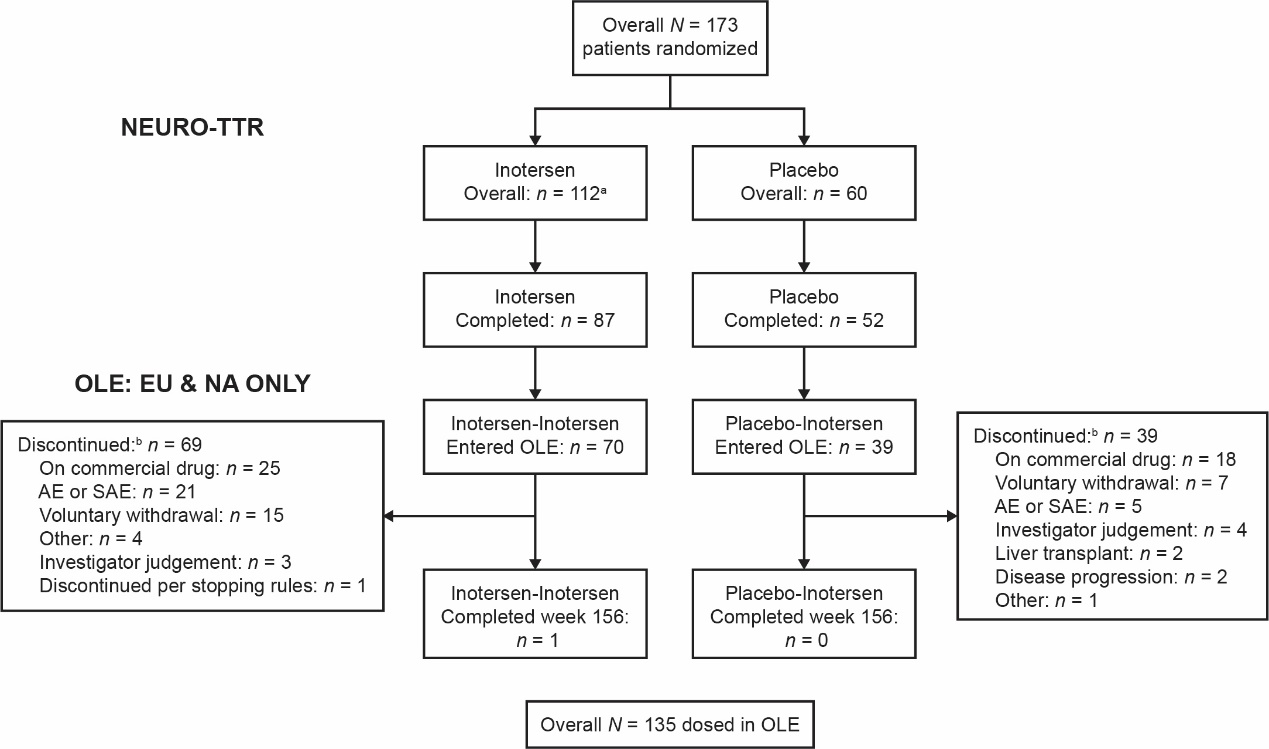


*AE* adverse event, *OLE* open-label extension, *SAE* serious adverse event

^a^One patient was randomly assigned in error and did not begin the trial regimen

^b^Primary reason for early treatment discontinuation

## Online Resource 2.

## Summary of treatment exposure^a^

| **Event, n (%)** | **Inotersen-inotersen**  **(*n* = 70)** | **Placebo-inotersen**  **(*n* = 39)** |
| --- | --- | --- |
| Dose interruption^b^ | 59 (84.3) | 36 (92.3) |
| Dose interruption due to platelet counts < 75 × 10^3^ /µL, *n* (%) | 13 (18.6) | 10 (25.6) |
| Dose interruption due to investigator/medical monitor discretion related to platelets, *n* (%) | 23 (32.9) | 16 (41.0) |
| Dose interruption due to investigator/medical monitor discretion related to renal events, *n* (%) | 5 (7.1) | 6 (15.4) |

^a^Represents all patients from NEURO-TTR open-label extension
^b^ ≥ 1 missed dose

## **Online Resource 3.**

## **Fig. 1** Proportion of patients that showed improvement over time in(A) the Modified Neuropathy Impairment Score +7 Neurophysiological Tests Composite Score (mNIS+7); (B) the Norfolk Quality of Life–Diabetic Neuropathy Questionnaire Total Score (QoL-DN); and (C) the 36-Item Short-Form Health Survey, version 2 (SF-36), Physical Component Summary score (PCS). Data shown are for the Europe and North America cohort enrolled in NEURO-TTR open-label extension. In general, the maximum screening period in the OLE study was 4 weeks. For simplicity, OLE baseline on the graph is noted as occurring 4 weeks after the end of the NEURO-TTR study


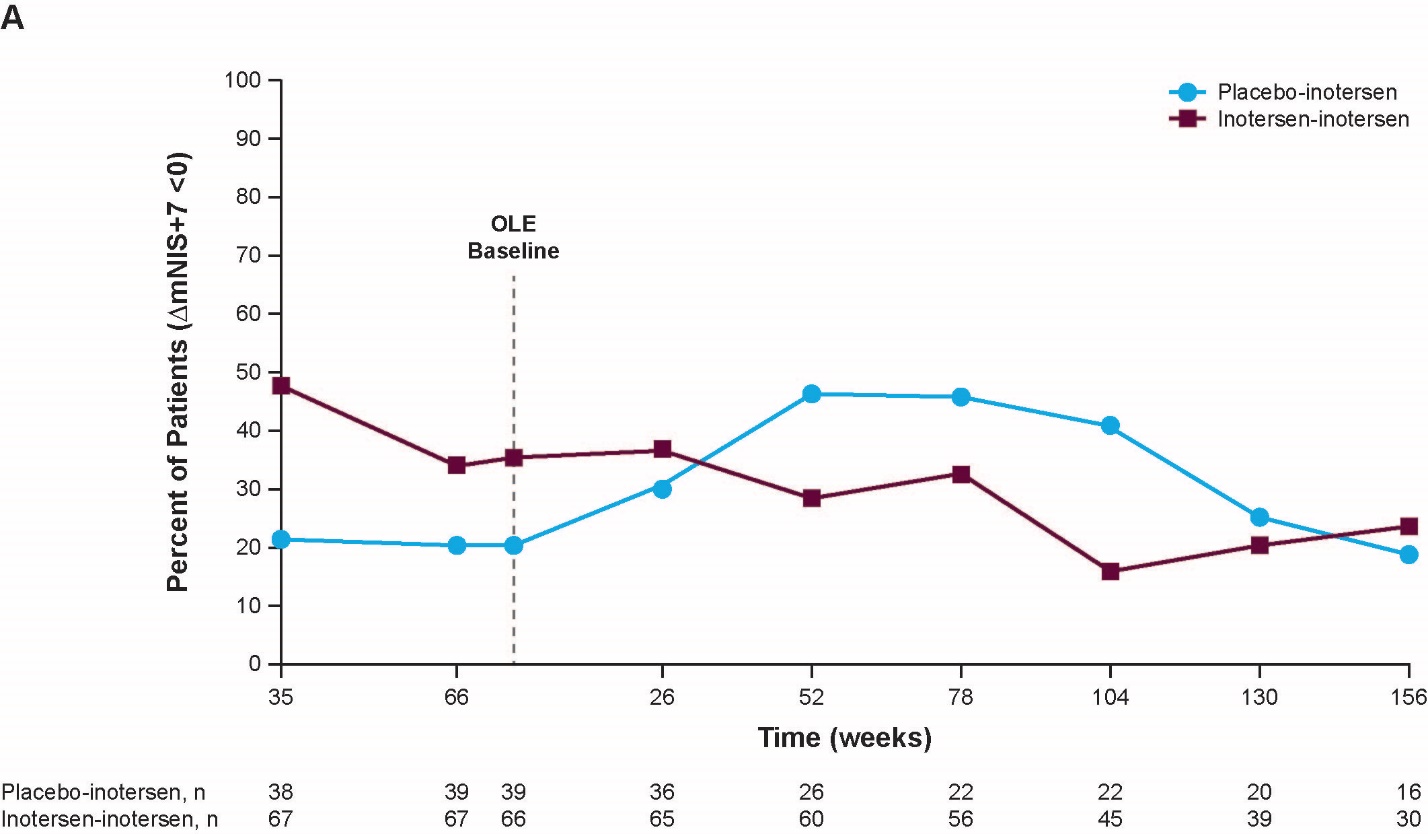


For the inotersen-inotersen group, improvement is indicated by the proportion of patients at all visits with a < 0-point change from NEURO-TTR baseline. For the placebo-inotersen group, improvement at NEURO-TTR week 35 and week 66 visits and OLE baseline visits are based on change < 0 points from NEURO-TTR baseline, while improvement at all post-baseline OLE visits are based on change < 0 points from OLE baseline


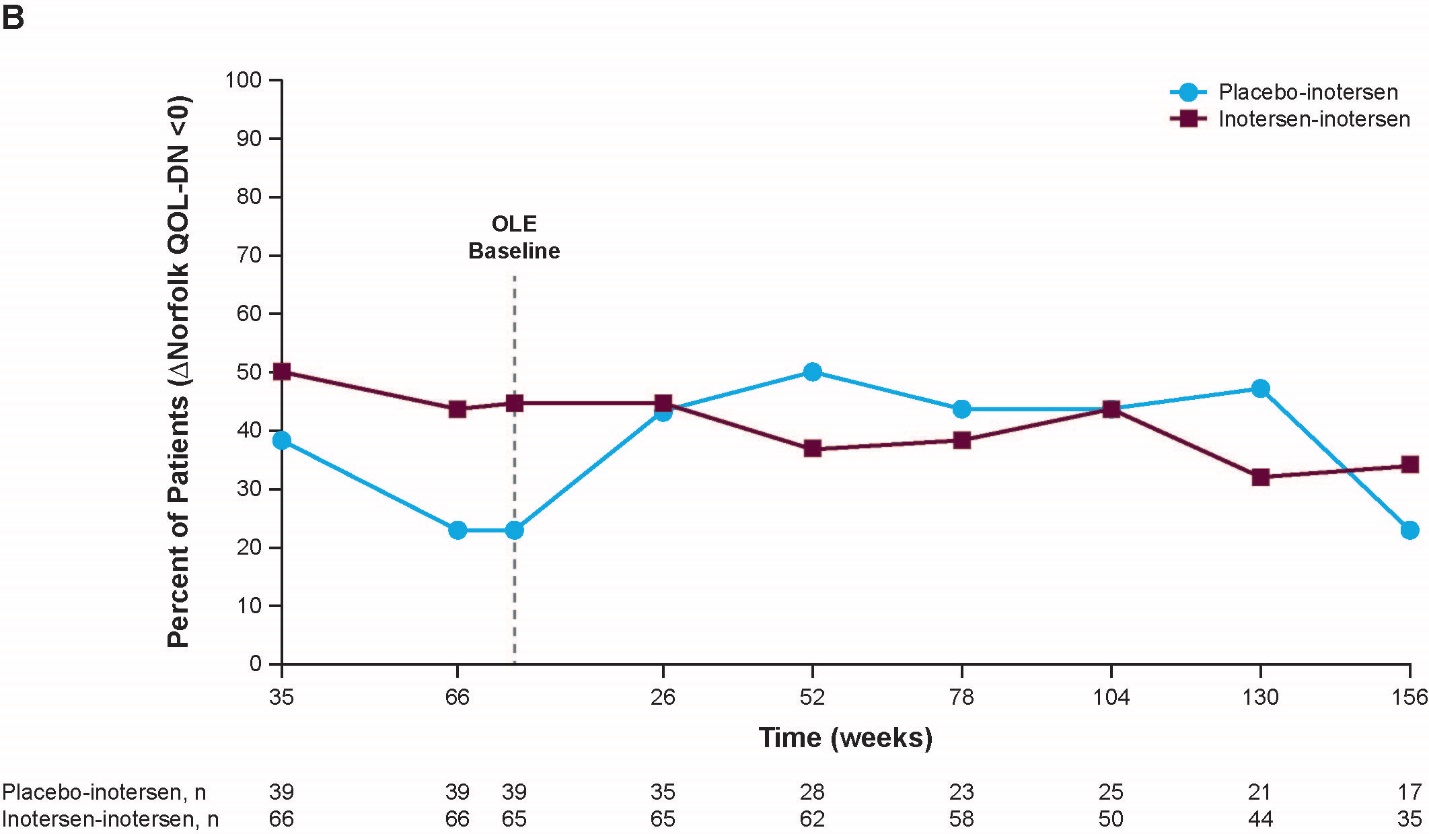


For the inotersen-inotersen group, improvement is indicated by the proportion of patients at all visits with a < 0-point change from NEURO-TTR baseline. For the placebo-inotersen group, improvement at NEURO-TTR week 35 and week 66 visits and OLE baseline visits are based on change < 0 points from NEURO-TTR baseline, while improvement at all post-baseline OLE visits are based on change < 0 points from OLE baseline


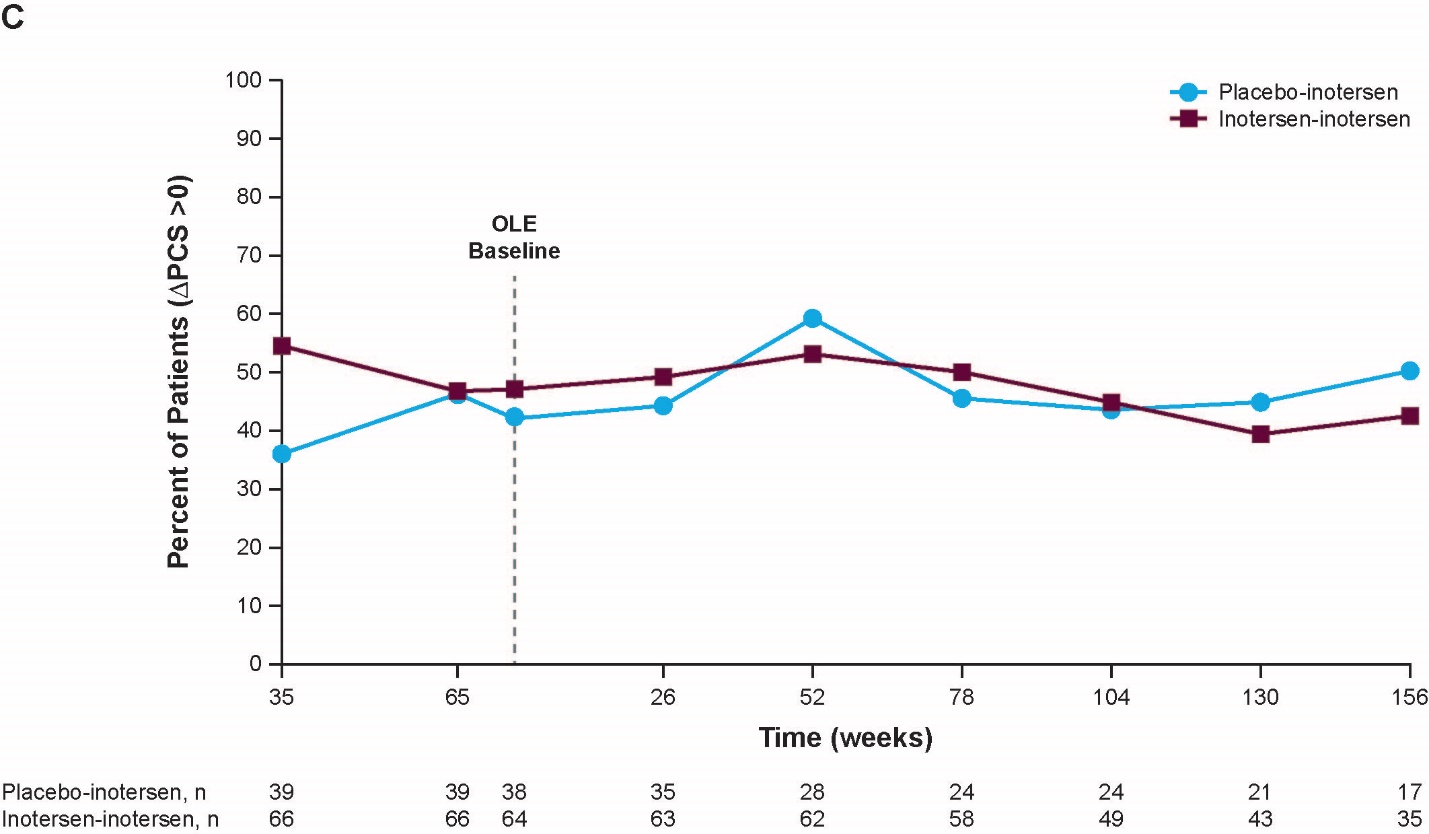


For the inotersen-inotersen group, improvement is indicated by the proportion of patients at all visits with a > 0-point change from NEURO-TTR baseline. For the placebo-inotersen group, improvement at NEURO-TTR week 35 and week 65 visits and OLE baseline visits are based on change > 0 points from NEURO-TTR baseline, while improvement at all post-baseline OLE visits are based on change > 0 points from OLE baseline

*OLE*, open-label extension

## **Online Resource 4.**

## **Fig. 2** Mean change from NEURO-TTR baseline to OLE week 156 in efficacy measures according to disease stage

Mean (±SE) change from NEURO-TTR baseline in (A) the Modified Neuropathy Impairment Score +7 Neurophysiological Tests Composite Score (mNIS+7); (B) the Norfolk Quality of Life–Diabetic Neuropathy Questionnaire Total Score (QoL-DN); (C) the 36-Item Short-Form Health Survey, version 2 (SF-36), Physical Component Summary score (PCS). Data shown are for all enrolled patients who received ≥ 1 dose of inotersen in the OLE and had ≥ 1 post-baseline efficacy assessment (full analysis set). The OLE baseline values were carried forward from the week 65 visit of the NEURO-TTR study for the SF-36 and from the week 66 visit for mNIS+7 and QoL-DN measures. FAP can be classified into three stages of disease based on ambulatory status: stage 1: do not require assistance with ambulation; stage 2: require assistance with ambulation; stage 3: wheelchair bound (Coutinho P, Martins da Silva A, Lopas L, J. (1980) Forty years of experience with type 1 amyloid neuropathy. Review of 483 cases. Amyloid and amyloidosis. Amsterdam, The Netherlands: Excerpta Medica; 1980: 88–98). In general, the maximum screening period in the OLE study was 4 weeks. For simplicity, OLE baseline on the graph is noted as occurring 4 weeks after the end of the NEURO-TTR study.

*OLE* open-label extension, *SE* standard error


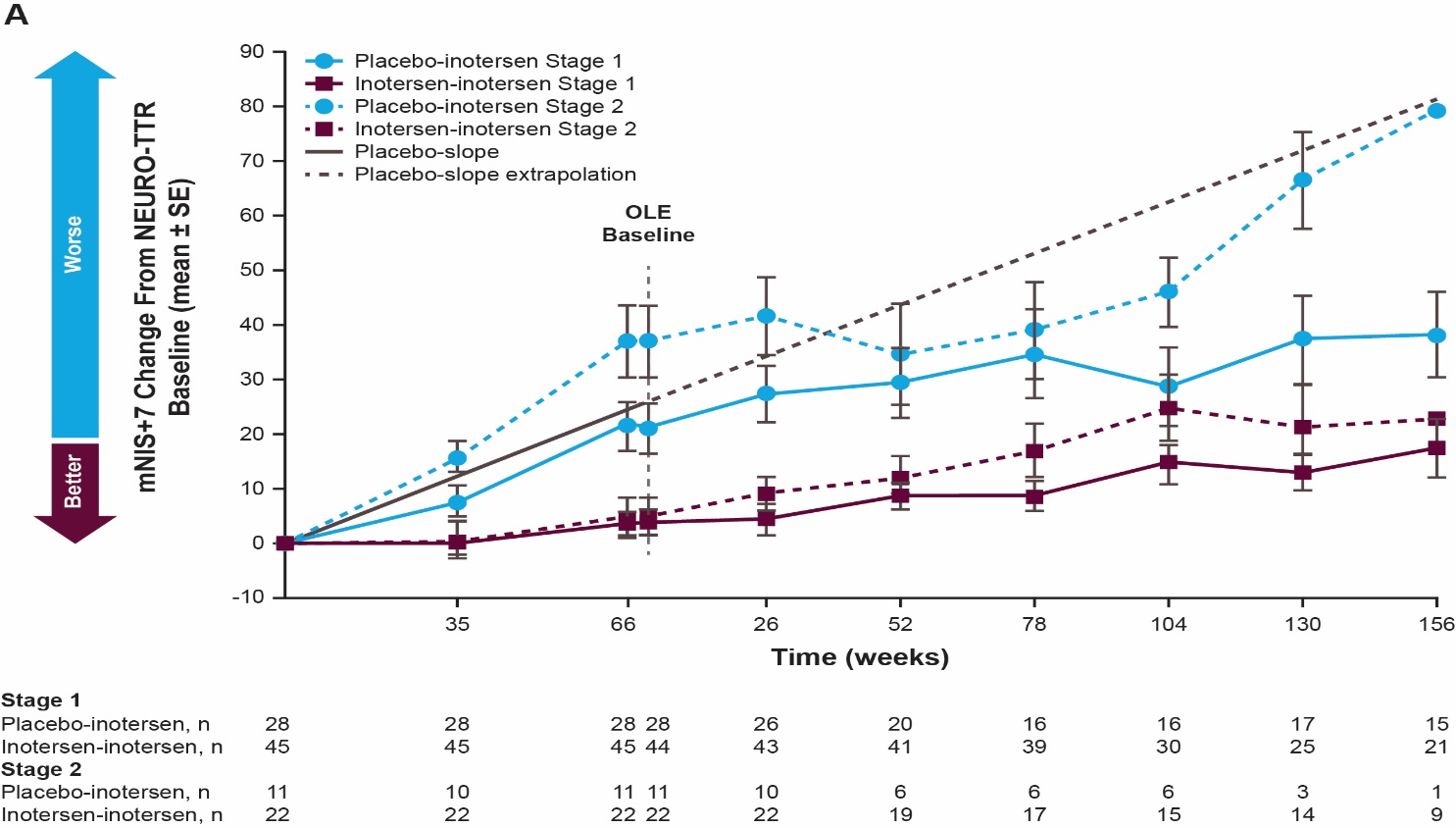


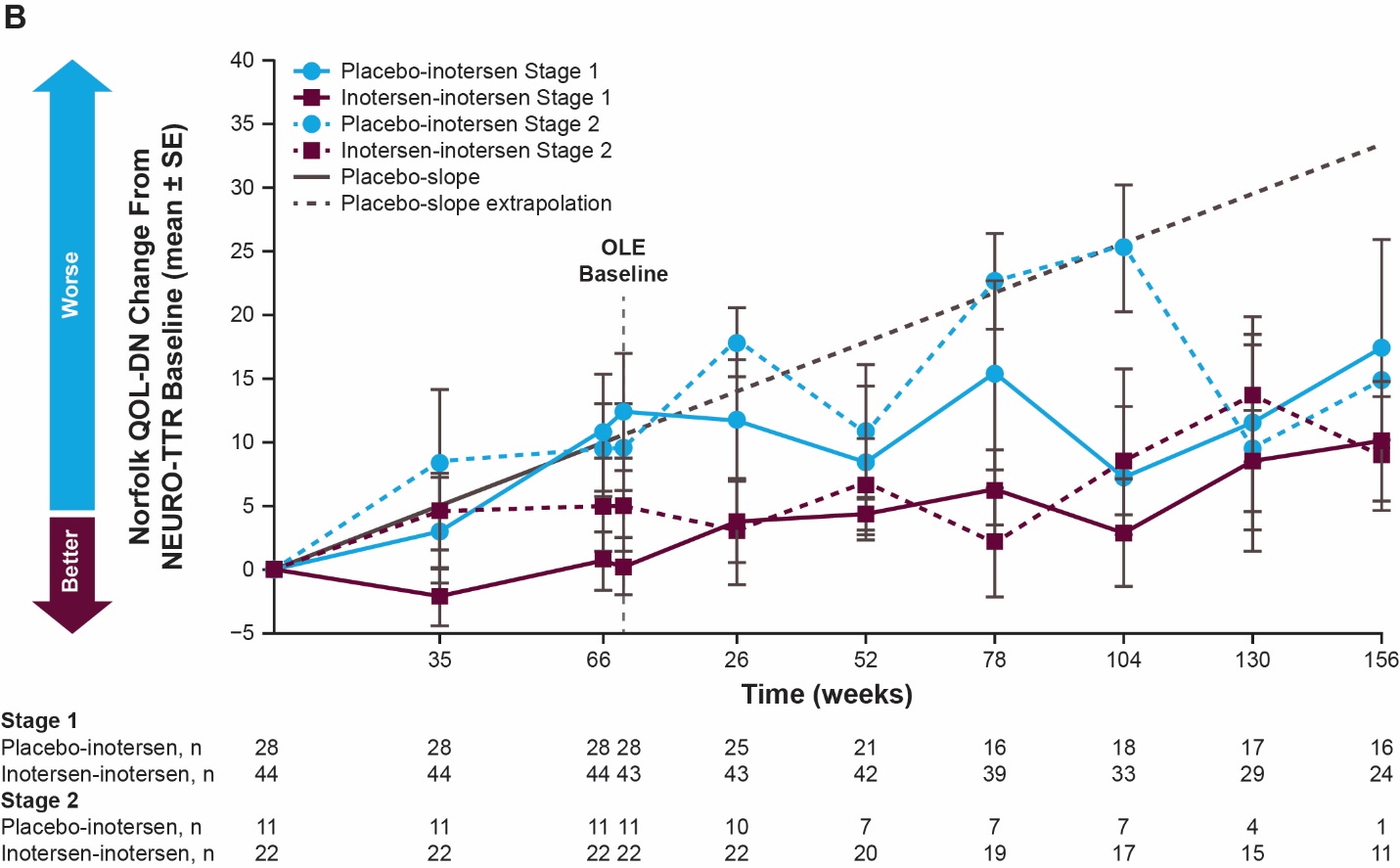


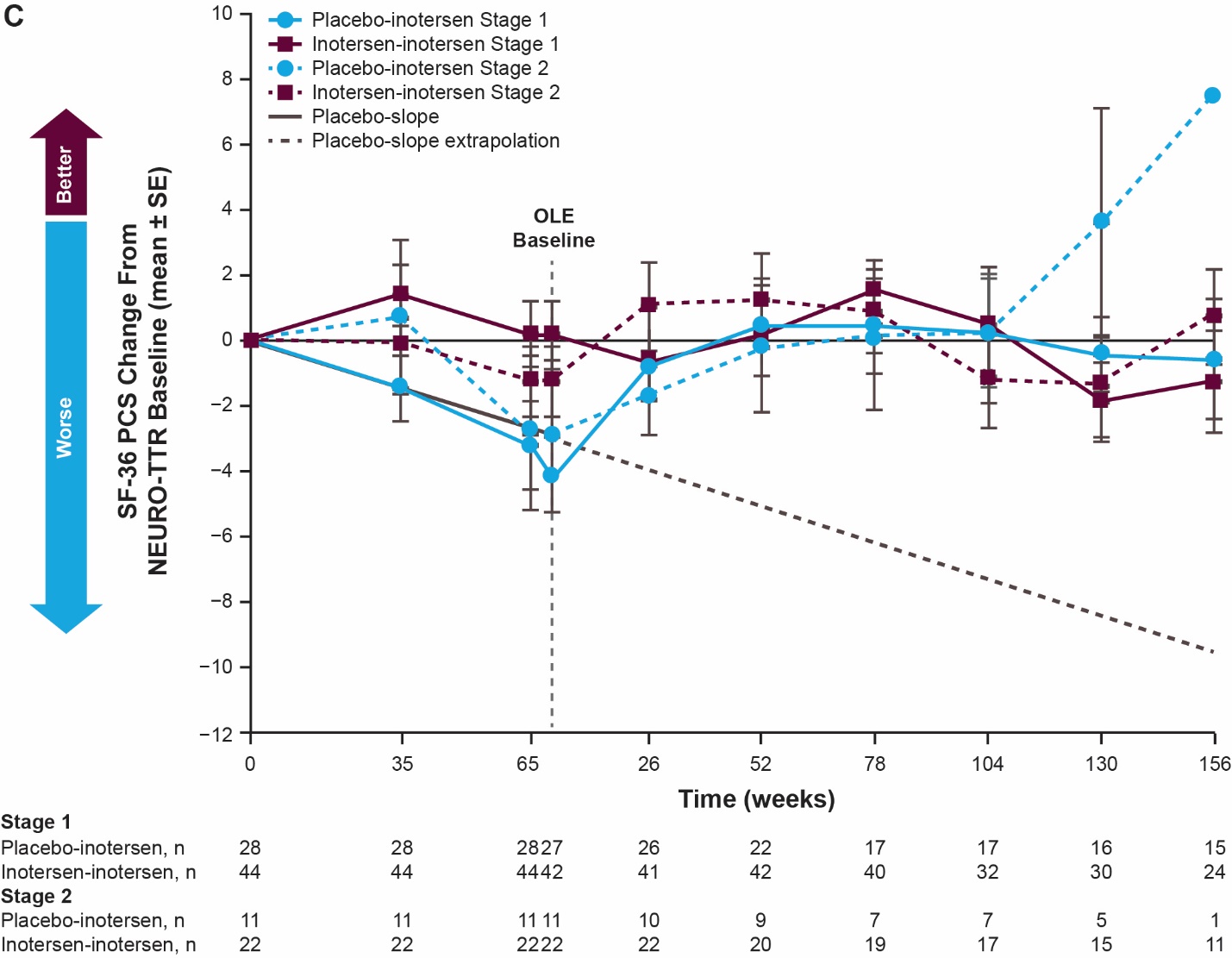


## **Fig. 3.** Mean change from NEURO-TTR baseline to OLE week 156 in efficacy measures according to V30M mutation status

Mean (±SE) change from NEURO-TTR baseline in (A) the Modified Neuropathy Impairment Score +7 Neurophysiological Tests Composite Score (mNIS+7); (B) the Norfolk Quality of Life–Diabetic Neuropathy Questionnaire Total Score (QoL-DN); (C) the 36-Item Short-Form Health Survey, version 2 (SF-36), Physical Component Summary score (PCS). Data shown are for all enrolled patients who received ≥ 1 dose of inotersen in the OLE and had ≥ 1 post-baseline efficacy assessment (full analysis set). The OLE baseline values were carried forward from the week 65 visit of the NEURO-TTR study for the SF-36 and from the week 66 visit for mNIS+7 and QoL-DN measures. In general, the maximum screening period in the OLE study was 4 weeks. For simplicity, OLE baseline on the graph is noted as occurring four weeks after the end of the NEURO-TTR study

*OLE* open-label extension, *SE* standard error


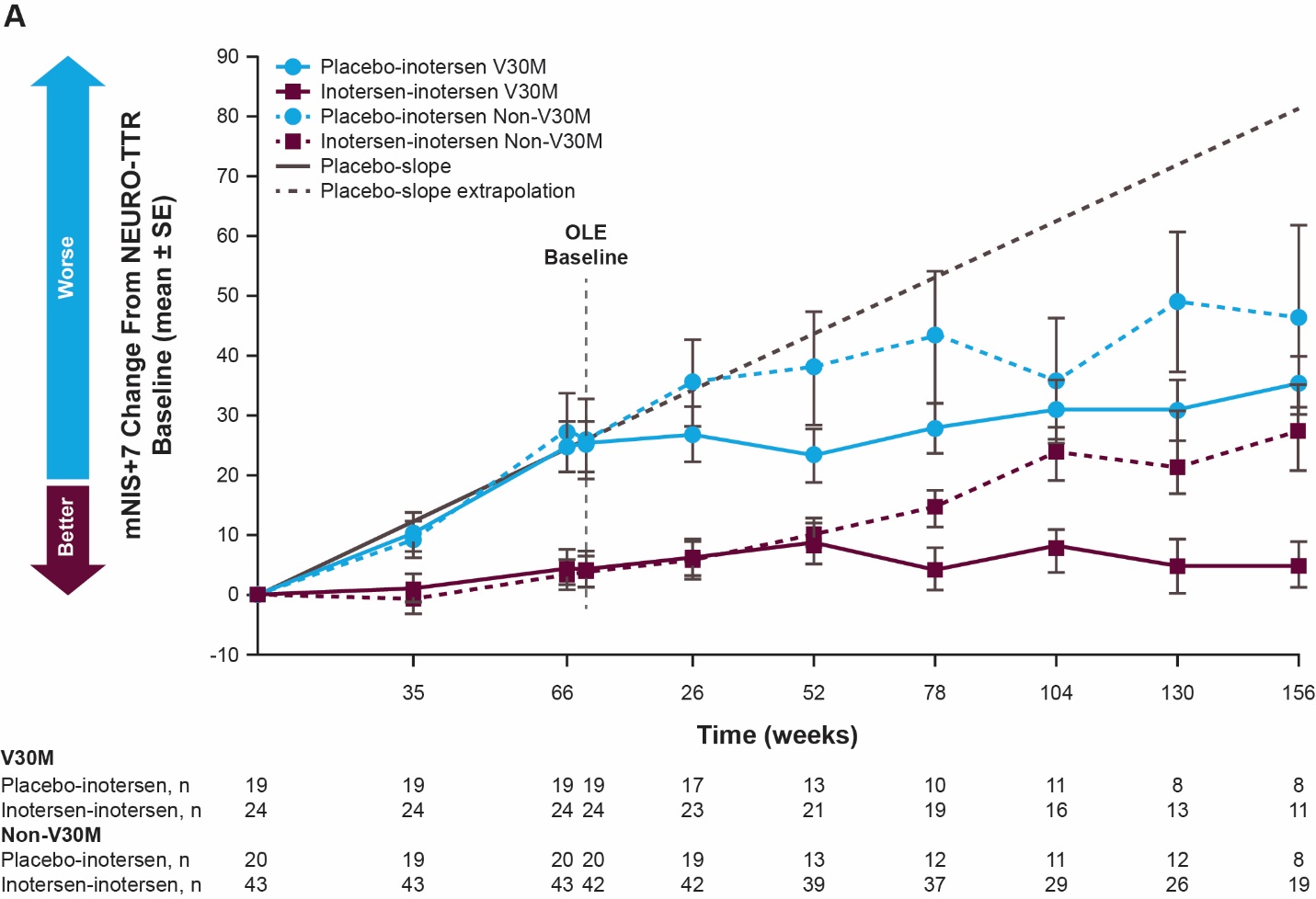


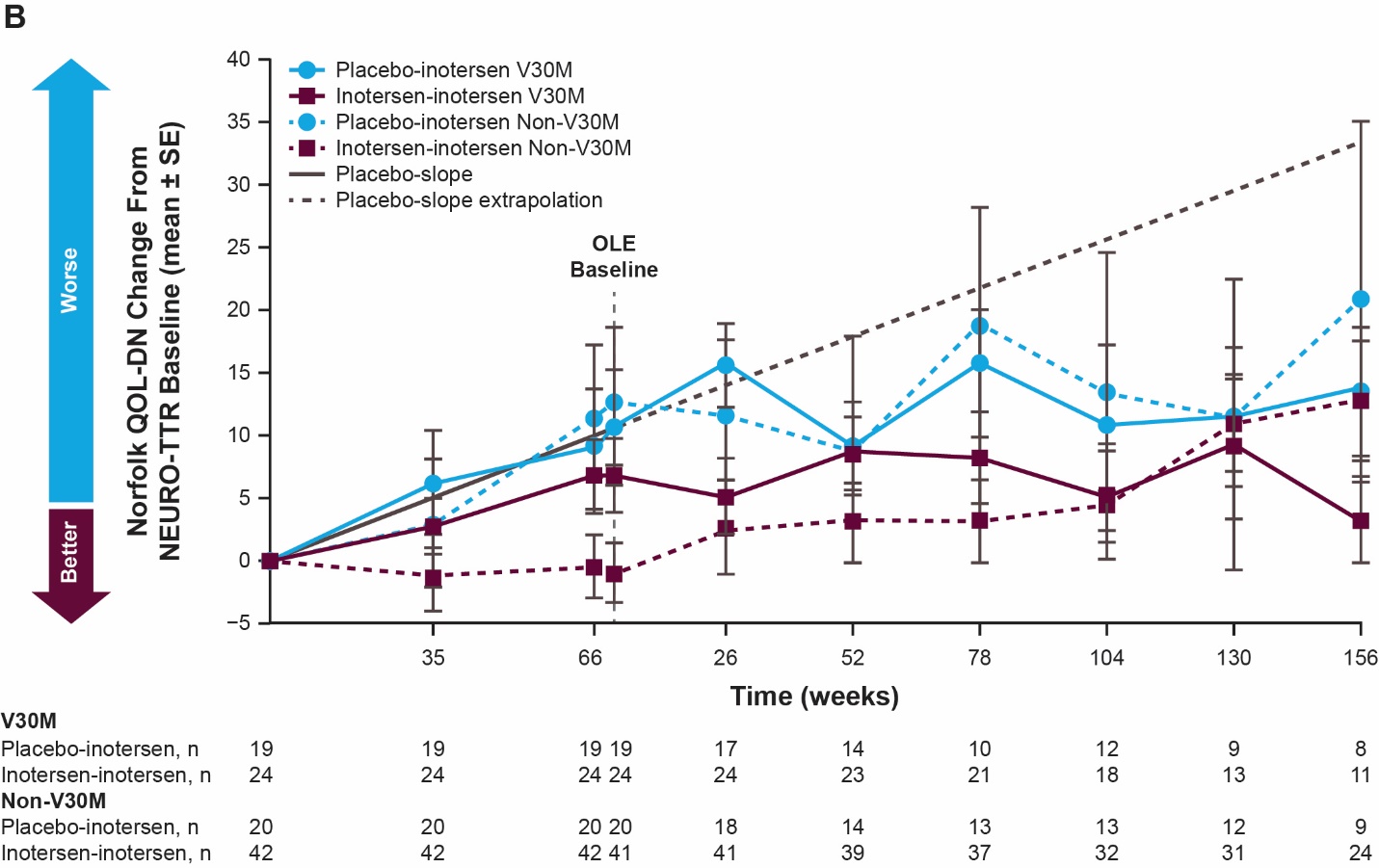


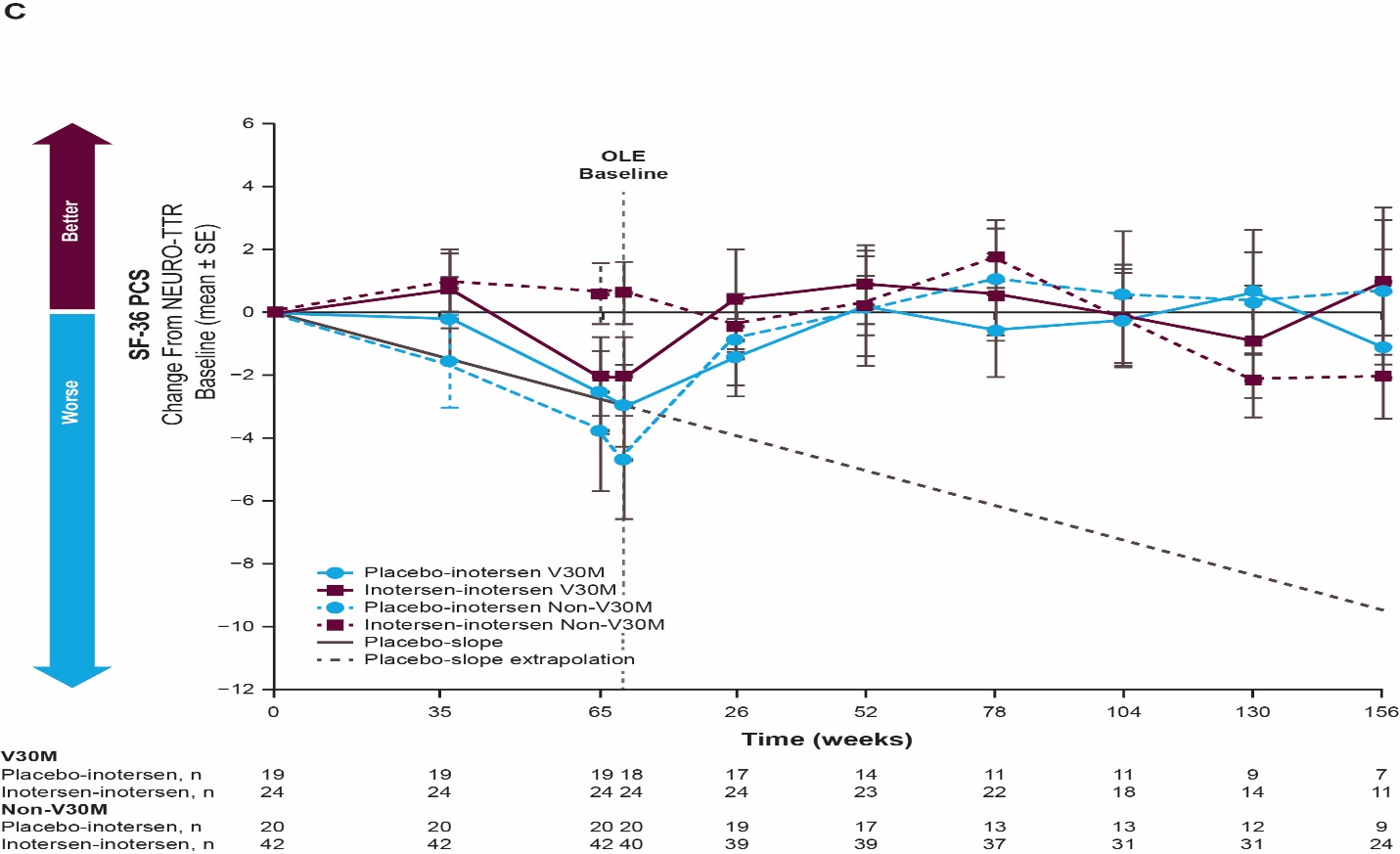


## **Fig. 4** Mean change from NEURO-TTR baseline to OLE week 156 in efficacy measures according to previous treatment with stabilisers

Mean (±SE) change from NEURO-TTR baseline in (A) the Modified Neuropathy Impairment Score +7 Neurophysiological Tests Composite Score (mNIS+7); (B) the Norfolk Quality of Life–Diabetic Neuropathy Questionnaire Total Score (QoL-DN); (C) the 36-Item Short-Form Health Survey, version 2 (SF-36), Physical Component Summary score (PCS). Data shown are for all enrolled patients who received ≥ 1 dose of inotersen in the OLE and had ≥ 1 post-baseline efficacy assessment (full analysis set). The OLE baseline values were carried forward from the week 65 visit of the NEURO-TTR study for the SF-36 and from the week 66 visit for mNIS+7 and QoL-DN measures. In general, the maximum screening period in the OLE study was 4 weeks. For simplicity, OLE baseline on the graph is noted as occurring 4 weeks after the end of the NEURO-TTR study

*OLE* open-label extension, *SE* standard error


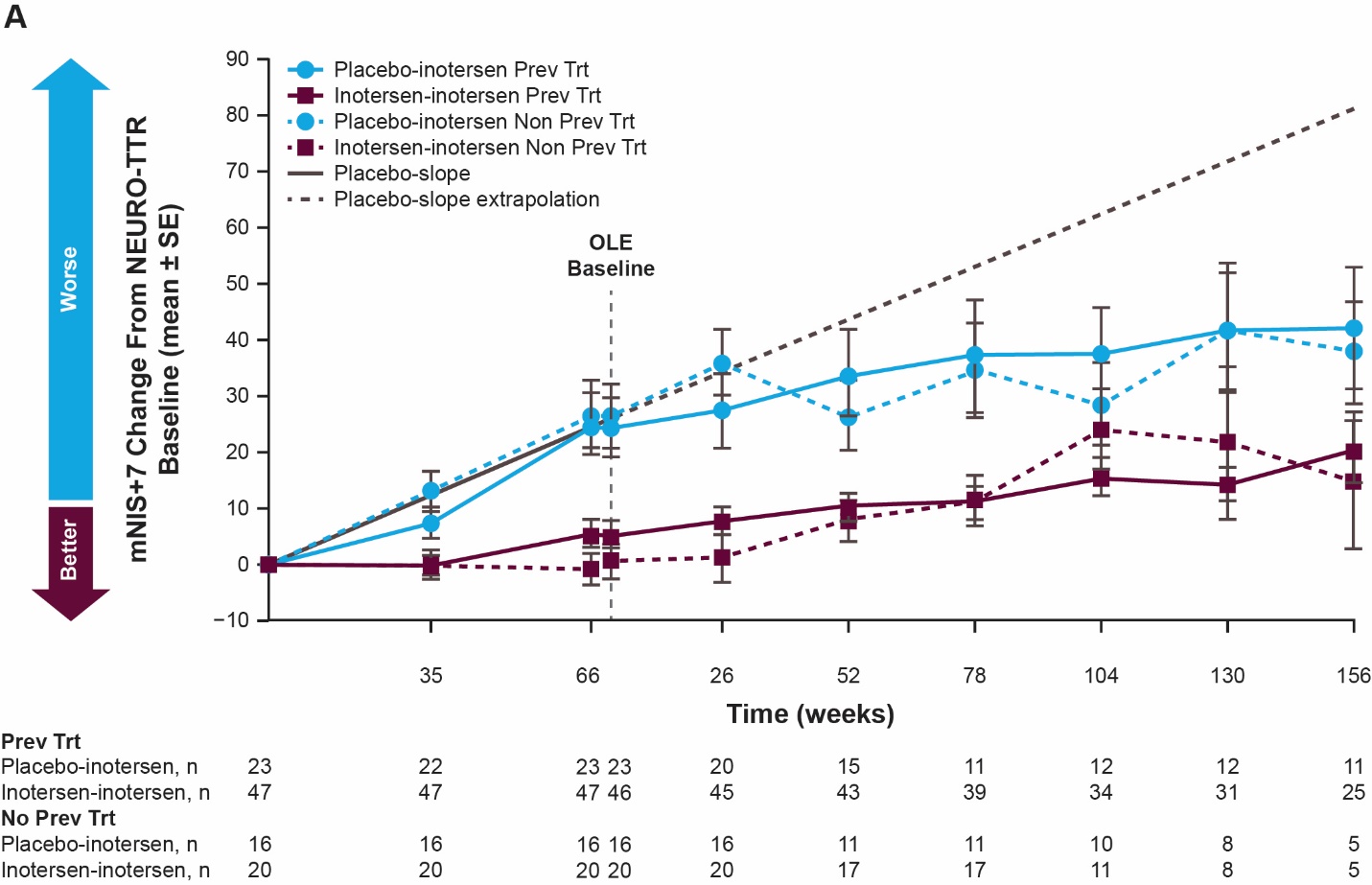


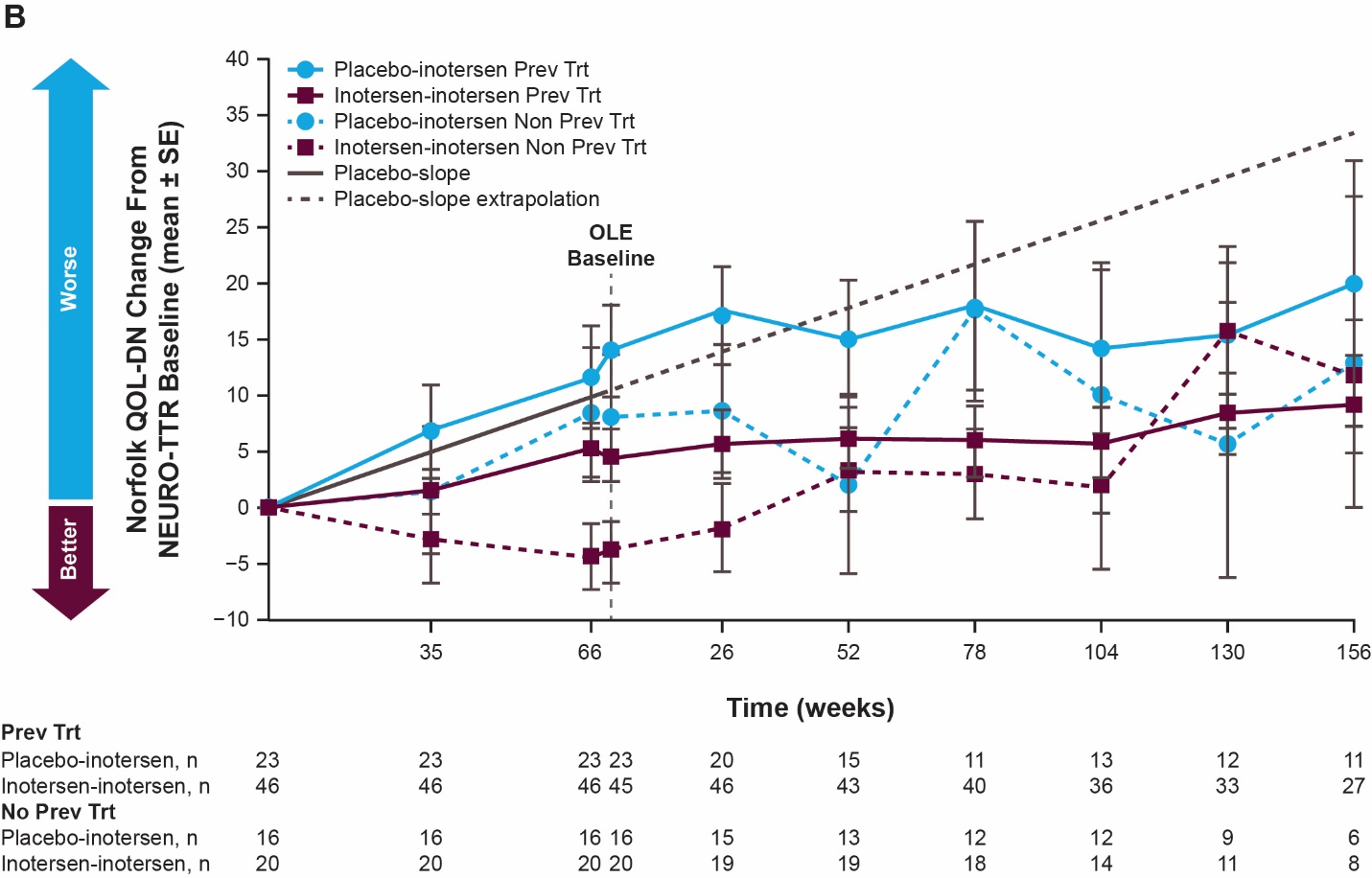


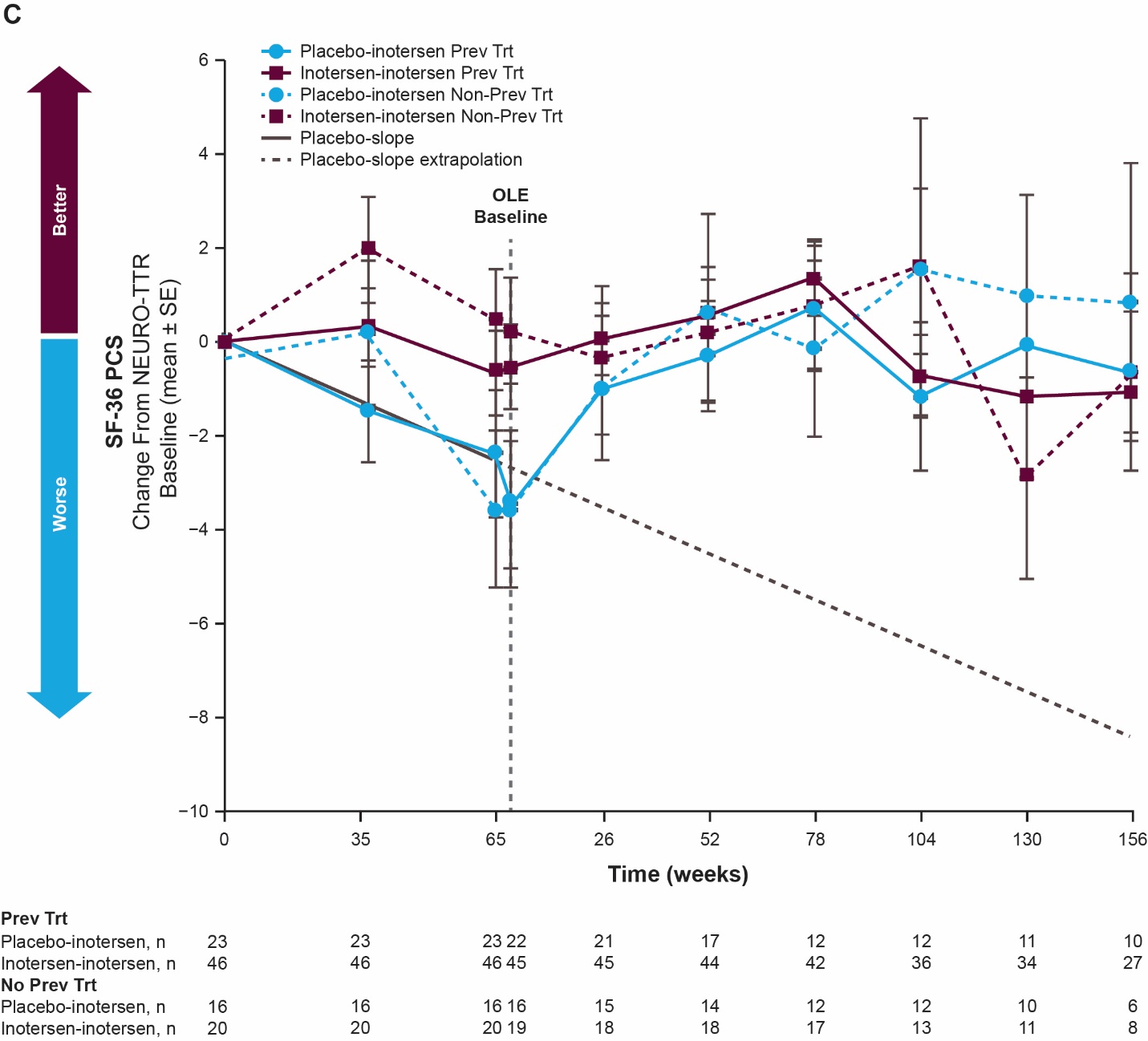


## **Online Resource 5**

## **Fig. 5** Maximum confirmed platelet count in the open-label extension study

Data shown are for the full safety dataset (*n* = 135), which comprises patients from Europe, North America, and Latin America/Australasia


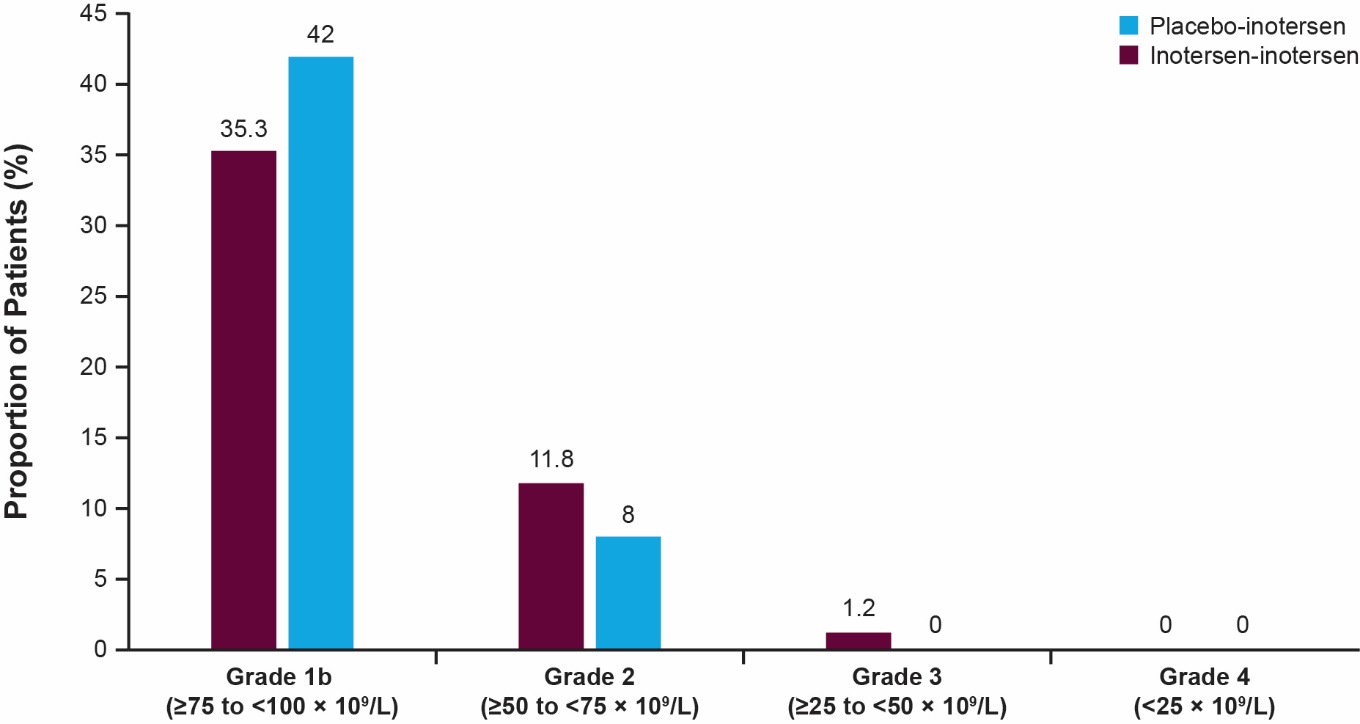


## **Online Resource 6**

**List of NEURO-TTR Open-Label Extension Investigators**

**Principal Investigators**

| **Last Name** | **First Name** | **Country** | **Institution** |
| --- | --- | --- | --- |
| Adams | David | France | Chu Bicetre Aphp French Referral Center for Fap/ Cornamyl Network |
| Barroso | Fabio | Argentina | Fleni (Fundacion Lucha Contra Las Enfermedades Neurologicas Infantiles) |
| Benson | Merrill | United States | Indiana University School of Medicine |
| Berk | John | United States | Boston University Medical Center |
| Brannagan | Thomas | United States | The Neurological Institute at Columbia University Medical Center |
| Campistol Plana | Josep Maria | Spain | Institut Clinic de Nefrologia i Urologia - ICNU, Hospital Clinic i Provincial de Barcelona |
| Gamez | Jose | Spain | Neurology Department, GMA Clinic, Autonomous University of Barcelona, and European Reference Network on Rare Neuromuscular Diseases (ERN EURO-NMD) |
| Coelho | Teresa | Portugal | Unidade Clinica de Paramiloidose-Centro Hospitalar Porto, EPE-Hospital Geral Santo Antonio |
| Conceicao | Isabel | Portugal | Centro Hospitalar Lisboa Norte - Hospital de Santa Maria |
| Cruz | Marcia Waddington | Brazil | Hospital Universitario Clementino Fraga Filho -HUCFF Universidade Federal do Rio de Janeiro |
| Drachman | Brian | United States | Penn Presbyterian Medical Center-University of Pennsylvania |
| Gane | Edward | New Zealand | New Zealand Liver Transplant Unit - Auckland City Hospital |
| Gertz | Morie | United States | Mayo Clinic |
| Gorevic | Peter | United States | Mount Sinai Medical Center |
| Heitner | Stephen | United States | Oregon Health & Science University-Knight Cardiovascular Institute |
| Kristen | Arnt | Germany | Medizinische Universtatsklinik Heidelberg |
| Merlini | Giampaolo | Italy | Centro Per Lo Studio E La Cura Delle Amiloidosi Sistemiche - Pavia - Fondazione Irccs Policlinico S.Matteo |
| Obici | Laura | Italy | Centro Per Lo Studio E La Cura Delle Amiloidosi Sistemiche - Pavia - Fondazione Irccs Policlinico S.Matteo |
| Plante-Bordeneuve | Violaine | France | Hopital Henri Mondor |
| Polydefkis | Michael | United States | The Johns Hopkins University (Jhu) - The Johns Hopkins Hospital (Jhh) |
| Salvi | Fabrizio | Italy | Irccs |
| Scheinberg | Morton | Brazil | Associacao de Assitencia a Crianca Deficiente (AACD) |
| Schmidt | Hartmut | Germany | Universitaetsklinikum Muenster |
| Souza Bulle Oliveira | Acary | Brazil | Universidade Federal de Sao Paulo |
| Vita | Giuseppe | Italy | Dip.Sc. Biomediche, Odont. e Imm.Funz.li, AOU Policlinico G. Martino |
| Wang | Annabel | United States | University of California Irvine (UCI) Health - Women's Healthcare Center & Center for Fetal Evaluation |
| Whelan | Carol | United Kingdom | University College London - National Amyloidosis Centre |

**Other Investigators**

| **Last Name** | **First Name** | **Country** | **Institution** |
| --- | --- | --- | --- |
| Abonour | Rafat | United States | Indiana University- Melvin and Bren Simon Cancer Center (IUSCC) |
| Alfonzo | Anna Lisa | Italy | Dip.Sc. Biomediche, Odont. e Imm.Funz.li, AOU Policlinico G. Martino |
| Al-Salameh | Abdallah | France | Hopital Kremlin APHP |
| Alves | Cristina | Portugal | Unidade Clinica de Paramiloidose-Centro Hospitalar Porto, EPE-Hospital Geral Santo Antonio |
| Ansell | Stephen | United States | Mayo Clinic |
| Antoniazzi | Elena | Italy | Centro Per Lo Studio E La Cura Delle Amiloidosi Sistemiche - Pavia - Fondazione Irccs Policlinico S.Matteo |
| Aragona | Pasquale | Italy | Dip.Sc. Biomediche, Odont. e Imm.Funz.li, AOU Policlinico G. Martino |
| Arrouasse | Raphaele | France | Hopital Henri Mondor |
| Azorin Contesse | Sebastian | Spain | Institut Clinic de Nefrologia i Urologia - ICNU, Hospital Clinic i Provincial de Barcelona |
| Barreau | Emmanuel | France | Hopital Kremlin APHP |
| Beaudonnet | Guillemette | France | Hopital Kremlin APHP |
| Becquemont | Laurent | France | Hopitaux Universitaires Paris Sud - Hopital Bicetre |
| Bremner | Fion | United Kingdom | University College London - National Amyloidosis Centre |
| Bressler | Neil M | United States | Johns Hopkins School Medical and Hospital |
| Brodie | Scott | United States | Icahn School of Medicine at Mount Sinai |
| Bruening-Missfelder | Ann-Christin | Germany | Universitaetsklinikum Muenster |
| Buadi | Francis | United States | Mayo Clinic |
| Cardoso Berensztejn | Amanda | Brazil | Hospital Universitario Clementino Fraga Filho -HUCFF Universidade Federal do Rio de Janeiro |
| Case | Jane | United States | Mayo Clinic |
| Cash | Tiyonnoh | United States | University of California Irvine (UCI) Health - Women's Healthcare Center & Center for Fetal Evaluation |
| Cauquil | Cecile | France | Hopital Bicetre - Paris Sud |
| Chaudhry | Vinay | United States | The Johns Hopkins University (Jhu) - The Johns Hopkins Hospital (Jhh) |
| Cicinnati | Vito | Germany | Universitaetsklinikum Muenster |
| Cortese | Andrea | Italy | Centro Per Lo Studio E La Cura Delle Amiloidosi Sistemiche - Pavia - Fondazione Irccs Policlinico S.Matteo |
| Coutinho | Conceicao | Portugal | Centro Hospitalar Lisboa Norte - Hospital de Santa Maria |
| Cucinotta | Francescopaolo | Italy | Aou Policlinico G. Martino-U.O. Di Neurologia E Malattie Neuromuscolari |
| Damy | Thibaud | France | Hopital Henri Mondor |
| Dasgupta | Noel | United States | Indiana University School of Medicine |
| de Andrade Guedes | Mariana | Brazil | Unidade de Pesquisa Clinica / Hospital Universitario Clementino Fraga Filho / UFRJ |
| Di Bella | Gianluca | Italy | Aou Policlinico G. Martino-U.O. Di Neurologia E Malattie Neuromuscolari |
| Dias da Silva | Moises | Brazil | Hospital Universitario Clementino Fraga Filho -HUCFF Universidade Federal do Rio de Janeiro |
| Dingli | David | United States | Mayo Clinic |
| Dos Santos Neto | Denizart | Brazil | Universidade Federal de Sao Paulo - UNIFESP / Hospital Sao Paulo Hospital de Ensino da UNIFESP Sao Paulo |
| Dyck | James | United States | Mayo Clinic |
| Eliahou | Ludivine | France | Hopital Antoine Beclere |
| Farhad | Khosro | United States | Columbia University Medical Center |
| Ferreira | Joao | Portugal | Centro Hospitalar Lisboa Norte (CHLN) EPE - Hospital de Santa Maria |
| Ferreira | Natalia | Portugal | Hospital Geral De Santo Antonio |
| Salvado | Maria | Spain | Hospital Universitari Vall d'Hebron |
| Fonder | Amie | United States | Mayo Clinic |
| Fontana | Marianna | United Kingdom | University College London - National Amyloidosis Centre |
| Gangat | Naseema | United States | Mayo Clinic |
| Gentile | Luca | Italy | Aou Policlinico G. Martino-U.O. Di Neurologia E Malattie Neuromuscolari |
| Gervais de Santa Rosa | Renata | Brazil | Hospital Universitario Clementino Fraga Filho -HUCFF Universidade Federal do Rio de Janeiro |
| Gillmore | Julian | United Kingdom | National Amyloidosis Centre-Royal Free Hospital-UCL Medical School |
| Go | Ronald | United States | Mayo Clinic |
| Goldman | Martin | United States | Icahn School of Medicine at Mount Sinai |
| Golmeia | Ricardo Prado | Brazil | AACD- Lar Escola\ Associacao de Assistencia a Crianca Deficiente |
| Gonsalves | Wilson | United States | Mayo Clinic |
| Goyal | Namita | United States | University of California Irvine (UCI) Health - Women's Healthcare Center & Center for Fetal Evaluation |
| Graham | Kellie | United States | Oregon Health & Science University-Knight Cardiovascular Institute |
| Hayman | Suzanne | United States | Mayo Clinic |
| Haymman-Gawrilow | Patricia | France | Centre Hospitalier Intercommunal de Creteil (CHIC) - Centre de ressources et de competences pour la mucoviscidose (C.R.C.M.) |
| Hebl | Victoria | United States | Oregon Health & Science University (OHSU) |
| Hobbs | Miriam | United States | Mayo Clinic |
| Hugenberg | Steven | United States | Indiana University School of Medicine (Non-IP Study supply address) |
| Imberti | Roberto | Italy | Amyloidosis Research & Treatment Center, Fondazione Irccs Policlinico San Matteo |
| Jonet De Azevedo Coutinho | Maria Da Conceicao | Portugal | Centro Hospitalar Lisboa Norte (CHLN) EPE - Hospital de Santa Maria |
| Joseph | Xavier | France | Hopital Henri Mondor |
| Judge | Daniel | United States | Johns Hopkins University-Center for Inherited Heart Disease |
| Kabar | Iyad | Germany | Universitaetsklinikum Muenster |
| Kapoor | Prashant | United States | Mayo Clinic |
| Karam | Chafic | United States | Oregon Health & Science University (OHSU) |
| Khella | Sami | United States | Penn Presbyterian Medical Center-University of Pennsylvania |
| Khoury | Julie | United States | Oregon Health & Science University-Knight Cardiovascular Institute |
| Kincaid | John | United States | Indiana University School of Medicine |
| Klein | Christopher | United States | Mayo Clinic |
| Kleyman | Inna | United States | The Neurological Institute at Columbia University Medical Center |
| kourelis | Taxiarchis | United States | Mayo Clinic |
| Kumar | Shaji | United States | Mayo Clinic |
| Kumar | Suwen | United States | Oregon Health & Science University-Knight Cardiovascular Institute |
| Labeyrie | Celine | France | Hopital Kremlin APHP |
| Lachmann | Helen J | United Kingdom | Royal Free Hospital-Royal Free London NHS Foundation Trust |
| Lacy | Martha | United States | Mayo Clinic |
| Lautre | Andrea | Argentina | Fleni |
| Le Tien | Valerie | France | Centre Hospitalier Intercommunal de Creteil (CHIC) - Centre de ressources et de competences pour la mucoviscidose (C.R.C.M.) |
| Lecoq | Anne Lise | France | Chu Bicetre Aphp French Referral Center for Fap/ Cornamyl Network |
| Lecorvoisier | Philippe | France | Hopital Henri Mondor |
| Leung | Nelson | United States | Mayo Clinic |
| Lin | Yi | United States | Mayo Clinic |
| Luiz Escorcio Bezerra | Marcio | Brazil | Associacao de Assitencia a Crianca Deficiente (AACD) |
| Mahmood | Shameem | United Kingdom | University College London - National Amyloidosis Centre |
| Marques-Neves | Carlos | Portugal | Centro Hospitalar Lisboa Norte - Hospital de Santa Maria |
| Martins Da Silva | Ana | Portugal | Hospital Geral De Santo Antonio |
| Mauermann | Michelle | United States | Mayo Clinic |
| Maurer | Mathew S | United States | Columbia University Medical Center |
| Mazzeo | Anna | Italy | Aou Policlinico G. Martino-U.O. Di Neurologia E Malattie Neuromuscolari |
| Mindel | Joel | United States | Icahn School of Medicine at Mount Sinai |
| Leitch | Megan M | United States | Columbia University Medical Center |
| Mozaffar | Tahseen | United States | University of California Irvine (UCI) Health - Women's Healthcare Center & Center for Fetal Evaluation |
| Muccioli | Cristina | Brazil | Universidade Federal de Sao Paulo - UNIFESP |
| Neves Cardoso | Marcio | Portugal | Unidade Clinica de Paramiloidose-Centro Hospitalar Porto, EPE-Hospital Geral Santo Antonio |
| Ng | Jason | United States | University of California Irvine (UCI) Health - Women's Healthcare Center & Center for Fetal Evaluation |
| Not | Adeline | France | Hopital Kremlin APHP |
| Orellana | Lucas | Argentina | Fleni |
| Pedrosa | Roberto | Brazil | Hospital Universitario Clementino Fraga Filho -HUCFF Universidade Federal do Rio de Janeiro |
| Pennesi | Mark | United States | Oregon Health & Science University-Knight Cardiovascular Institute |
| Perlini | Stefano | Italy | Centro Per Lo Studio E La Cura Delle Amiloidosi Sistemiche - Pavia - Fondazione Irccs Policlinico S.Matteo |
| Pimentel Fonseca Golmia | Andrea | Brazil | AACD- Lar Escola\ Associacao de Assistencia a Crianca Deficiente |
| Pogoda | Christian | Germany | Universitaetsklinikum Muenster |
| Postorino | Elisa Imelde | Italy | Dip.Sc. Biomediche, Odont. e Imm.Funz.li, AOU Policlinico G. Martino |
| Quarta | Candida | United Kingdom | University College London - National Amyloidosis Centre |
| Rajkumar | Sundararajan | United States | Mayo Clinic |
| Rania | Laura | Italy | Dip.Sc. Biomediche, Odont. e Imm.Funz.li, AOU Policlinico G. Martino |
| Ravi | Sriram | United States | Knight Cardiovascular Institute-Oregon Health & Science University |
| Reis | Antonio | Portugal | Unidade Clinica de Paramiloidose-Centro Hospitalar Porto, EPE-Hospital Geral Santo Antonio |
| Rezk | Tamer | United Kingdom | University College London - National Amyloidosis Centre |
| Rizzo | Vincenzo | Italy | Dip.Sc. Biomediche, Odont. e Imm.Funz.li, AOU Policlinico G. Martino |
| Robinson-Papp | Jessica | United States | Icahn School of Medicine at Mount Sinai |
| Robson | Anthony | United Kingdom | University College London - National Amyloidosis Centre |
| Rocha Pinto | Luis Felipe | Brazil | Hospital Universitário Clementino Fraga Filho - HUCFF |
| Rocha Pinto | MarcusVinicius | Brazil | Hospital Universitario Clementino Fraga Filho -HUCFF Universidade Federal do Rio de Janeiro |
| Rodas | Lida | Spain | Hospital Clinic de Barcelona - Institut Clinic de Malalties Hematologiques i Oncologiques (ICMHO) |
| Rousseau | Antoine | France | Hopital Kremlin APHP |
| Roy | Gulmohor | United States | University of California Irvine (UCI) Health - Women's Healthcare Center & Center for Fetal Evaluation |
| Ruberto | Giulio | Italy | Centro Per Lo Studio E La Cura Delle Amiloidosi Sistemiche - Pavia - Fondazione Irccs Policlinico S.Matteo |
| Russell | Stephen | United States | Mayo Clinic |
| Russell | Stuart | United States | The Johns Hopkins University (Jhu) - The Johns Hopkins Hospital (Jhh) |
| Russo | Massimo | Italy | Aou Policlinico Di Messina |
| Ruzhansky | Katherine | United States | The Neurological Institute at Columbia University Medical Center |
| Salhi | Hayet | France | Hopital Henri Mondor |
| Santos | Miguel | Portugal | Centro Hospitalar Lisboa Norte, EPE Hospital Santa Maria |
| Schilling | Matthias | Germany | Universitaetsklinikum Muenster |
| Scholl | Hendrik P N | United States | The Johns Hopkins University (Jhu) - The Johns Hopkins Hospital (Jhh) |
| Shaath | Hussain | United States | The Neurological Institute at Columbia University Medical Center |
| Shah | Syed Mahmood | United States | The Johns Hopkins University (Jhu) -Wilmer Eye Institute |
| Siddiqui | Mustaqeem | United States | Mayo Clinic |
| Singh | Mandeep | United States | Wilmer Eye Institute |
| Slama | Michel | France | Hopital Antoine Beclere |
| Soueid | Eric | France | Centre Hospitalier Intercommunal de Creteil (CHIC) - Centre de ressources et de competences pour la mucoviscidose (C.R.C.M.) |
| Staff | Nathan | United States | Mayo Clinic |
| Stancanelli | Claudia | Italy | AOU Policlinico G. Martino - UOC di Neurologia e Malattie Neuromuscolari |
| Statescu | Claudius | France | Hopital Antoine Beclere |
| Thawani | Sujata | United States | The Neurological Institute at Columbia University Medical Center |
| Theaudin | Marie | France | Hopital Bicetre - Paris Sud |
| Torres Ferrus | Marta | Spain | Hospital Universitari Vall d'Hebron |
| Tournev | Ivailo | Bulgaria | Alexandrovska University Hospital |
| Tuleta | Izabela | Germany | Universitaetsklinikum Muenster |
| Ulane | Christina M | United States | The Neurological Institute at Columbia University Medical Center |
| Wajnsztajn | Fernanda | United States | Columbia University Medical Center |
| Weber | Peter | Germany | Universitaetsklinikum Muenster |
| Wechalekar | Ashutosh | United Kingdom | Universty College London Medical School |
| Weimer | Louis H | United States | The Neurological Institute at Columbia University Medical Center |
| Weis | Tobias | Germany | Universitaetsklinikum Muenster |
| Wiesman | Janice | United States | Boston University Amyloidosis Center |
| Yang | Paul | United States | Oregon Health & Science University-Knight Cardiovascular Institute |
| Youngstein | Taryn | United Kingdom | National Amyloidosis Centre-Royal Free Hospital-UCL Medical School |
| Zambrowski | Olivia | France | Centre Hospitalier Intercommunal de Creteil (CHIC) - Centre de ressources et de competences pour la mucoviscidose (C.R.C.M.) |
| Zeldenrust | Steven | United States | Mayo Clinic |
